# The epidemiologic and economic burden of dengue in Singapore: A systematic review

**DOI:** 10.1371/journal.pntd.0012240

**Published:** 2024-06-10

**Authors:** Rita Ting, Borame L. Dickens, Riona Hanley, Alex R. Cook, Ellyana Ismail

**Affiliations:** 1 Takeda Malaysia Sdn Bhd, Selangor, Malaysia; 2 Saw Swee Hock School of Public Health, National University of Singapore and National University Health System, Singapore, Singapore; 3 Takeda Pharmaceuticals International AG, Zurich, Switzerland; University of California Irvine, UNITED STATES

## Abstract

**Background:**

Despite its well-regarded vector control program, Singapore remains susceptible to dengue epidemics. To assist evaluation of dengue interventions, we aimed to synthesize current data on the epidemiologic and economic burden of dengue in Singapore.

**Methodology:**

We used multiple databases (PubMed, Embase, Cochrane, international/national repositories, surveillance) to search for published and gray literature (2000–2022). We included observational and cost studies, and two interventional studies, reporting Singapore-specific data on our co-primary outcomes, dengue incidence and dengue-related costs. Quality was assessed using the Newcastle-Ottawa Scale and an adapted cost-of-illness evaluation checklist. We performed a narrative synthesis and grouped studies according to reported outcomes and available stratified analyses.

**Findings:**

In total, 333 reports (330 epidemiological, 3 economic) were included. Most published epidemiological studies (89%) and all economic studies were of good quality. All gray literature reports were from the Ministry of Health or National Environment Agency. Based predominantly on surveillance data, Singapore experienced multiple outbreaks in 2000–2021, attaining peak incidence rate in 2020 (621.1 cases/100,000 person-years). Stratified analyses revealed the highest incidence rates in DENV-2 and DENV-3 serotypes and the 15–44 age group. Among dengue cases, the risk of hospitalization has been highest in the ≥45-year-old age groups while the risks of dengue hemorrhagic fever and death have generally been low (both <1%) for the last decade. Our search yielded limited data on deaths by age, severity, and infection type (primary, secondary, post-secondary). Seroprevalence (dengue immunoglobulin G) increases with age but has remained <50% in the general population. Comprising 21–63% indirect costs, dengue-related total costs were higher in 2010–2020 (SGD 148 million) versus the preceding decade (SGD 58–110 million).

**Conclusion:**

Despite abundant passive surveillance data, more stratified and up-to-date data on the epidemiologic and economic burden of dengue are warranted in Singapore to continuously assess prevention and management strategies.

## Author summary

Even with its well-regarded vector control program, Singapore has continued to experience dengue outbreaks in recent years. Evaluation of these dengue interventions requires updated information on disease distribution and its costs. Accordingly, we gathered 20 years’ worth of data in Singapore from several research databases. Dengue surveillance by Singapore’s Ministry of Health, the source of most data, showed that multiple epidemics occurred in 2000–2021, with a peak in 2020, although the prevailing type of dengue virus has shifted over time. Dengue mostly affects the youth (15–24 years of age) and middle-aged adults, but older adults are more frequently hospitalized. Nonetheless, severe disease and death are uncommon among those affected. In our search, we found only limited data on the influence of age, disease severity, or order of infection (first, second, or higher) on dengue deaths. The costs of dengue have increased in the last decade, as costs indirectly related to medical care remain significant cost contributors. Similarly, stratification of costs by age, epidemic versus non-epidemic years and societal or governmental perspective are presently lacking in local literature. More detailed data on the epidemiology and economic burden of dengue could facilitate the design of more tailored interventions to control dengue in Singapore.

## Introduction

Dengue, the fastest spreading mosquito-borne viral disease in the world, has increased in global incidence by 30-fold between 1960 and 2010 [1,2]. Annually, it now affects over 100 million individuals across the world [3]. Apart from a high burden of disease, it also imposes an economic burden from direct and indirect costs, incurring an estimated USD 8.9 billion in total costs globally in 2013 [4].

The dengue virus (DENV) has four distinct serotypes (DENV-1 to -4) and is transmitted via the tropical/subtropical mosquito vectors *Aedes aegypti* and *Aedes albopictus*, whose extrinsic incubation periods are influenced by ambient temperature and the climate [2]. Geographical areas with dengue transmission have expanded in recent years, mainly as a result of increased population growth rate, unplanned urbanization, inefficient mosquito control, climate change, and increased frequency of international travel from endemic to non-endemic regions [1,5,6].

Based on the World Health Organization (WHO) [2] regional classification, more than 70% of the world’s dengue at-risk population live in only two regions, including the Western Pacific Region. Meanwhile, according to the Global Burden of Disease (GBD) [7] grouping, the high-income Asia Pacific region exhibited the highest annual increment in dengue incidence from 1990 to 2019. In the same 20-year period, it was also the fourth leading region in terms of rise in disability-adjusted life years (DALYs) from dengue [6]. Singapore belongs to both these WHO- and GBD-defined regions.

The presence of mosquito vectors and appropriate environmental conditions for virus transmission makes dengue endemic in Singapore [8]. Nevertheless, the dengue control program in Singapore is one of the most highly regarded globally, effectively reducing the force of infection of dengue in the background of a progressively aging Singaporean population [9]. While vector control in Singapore applies an interagency “whole-of-government” approach, it primarily consists of environmental management, including more recent innovations such as (1) *Wolbachia* interventions, whereby eggs produced by male *Wolbachia*-infected mosquitoes and wild-type females do not hatch (“cytoplasmic incompatibility”); (2) the use of *Aedes aegypti* breeding percentages as risk indicators; and (3) risk mapping (e.g., spatiotemporal models) for targeted resource allocation [9].

Singapore’s vector control initiatives are complemented by its disease surveillance program. Medical practitioners and laboratories in Singapore are mandated to report a case of dengue fever or dengue hemorrhagic fever (DHF) soon after diagnosis [8,9]. Dengue is comprehensively monitored to understand not only its incidence by passive surveillance but also its serotype distribution by sentinel active surveillance. In the latter, random blood samples are shared for serotyping and genotyping to detect serotype shifts and provide an early warning of potential outbreaks [10]. Although definitions of dengue outbreak are highly heterogenous in the literature [11], for this paper it is defined as ‘a temporal anomaly in incidence rate from the expected number of cases for the period based on historical data’.

Despite dengue control efforts, Singapore has remained susceptible to epidemics, experiencing several outbreaks in the past decade [9]. This phenomenon has been attributed to high-level mobility in and out of Singapore, as well as low herd immunity due to successful reduction of disease transmission [9]. Meanwhile, the total costs of dengue are also exceptionally high in Singapore compared with other Southeast Asian countries, attributable to factors such as higher work absenteeism costs related to higher GDP per capita, and higher costs of hospitalization [12]. The latter costs are borne by a combination of the individual, private or national income tax-based insurance and the government, with the ratio of contributions dependent on patient eligibility for government subsidies [13]. Furthermore, the highly effective and rigorous publicly-funded vector control program implemented in Singapore comprises ~40–60% of total dengue-related costs [14].

Although the field of dengue vaccine production has advanced in recent years, challenges persist in terms of developing vaccines that are equally protective against all serotypes [15]. To choose among these multiple available interventions for dengue control, up-to-date and comprehensive information about dengue epidemiology and costs in Singapore are needed. Hence, we undertook this systematic review to synthesize currently available data on the epidemiologic and economic burden of dengue in Singapore.

## Methods

This review was conducted in line with the Cochrane Handbook for Systematic Reviews of Interventions [16], the Preferred Reporting Items for Systematic Reviews and Meta-Analysis (PRISMA) [17], and the Synthesis Without Meta-analysis reporting guidelines [18].

### Criteria for study inclusion

We included studies conducted in Singapore that involved individuals of any age with suspected dengue, confirmed dengue, or potential previous dengue exposure. We excluded international studies from which data exclusive to Singapore could not be extracted. Studies with an observational (real-world) study design (e.g., case-control, cohort, cross-sectional, ecological, genomic) and cost studies (e.g., cost description, cost analysis, cost-utility analysis, cost-effectiveness analysis) were eligible for inclusion. Interventional studies (e.g., clinical trials) were considered if usable baseline data were available. No restrictions were placed on the type of intervention or comparator. Case reports and systematic reviews without meta-analyses were excluded.

In terms of outcome measures, we included studies that reported dengue incidence in Singapore (co-primary outcome of interest) or other outcomes pertaining to epidemiologic burden: serotype distribution, seroprevalence, hospitalization, mortality, complications, and the lengths of hospitalization, treatment, illness, or disability. For economic burden, we considered studies reporting dengue-related total costs (co-primary outcome of interest) or disaggregated costs (i.e., direct medical, direct non-medical, indirect societal).

### Search methods

Using a predefined search strategy composed of a combination of controlled vocabulary and free-text terms (**S1 and S2 Tables**), we searched multiple electronic journal databases until 31 May 2022: Embase, MEDLINE via PubMed (1966–2022), and the Cochrane Library. Our search across journal databases was limited to publications in English and human studies. Additionally, to find both published data and gray literature, we searched several international and national research databases (**S3 Table**), including dengue surveillance databases: WHO Bulletins and Newsletters for Dengue Situation Updates, Ministry of Health (MOH) Singapore, and Singapore’s National Environment Agency (NEA).

A search was conducted separately for the epidemiologic burden (i.e., ‘epidemiology search’) and economic burden of dengue (i.e., ‘costs search’). To retrieve only the latest data, but also detect epidemiology trends over time and accommodate for long-term vector control and dengue prevention strategies, we set the search period to 2000–2022. Meanwhile, the search period for costs of dengue was restricted to 2009–2022 (the previous ~10 years), in accordance with the recommended approach to obtaining cost-of-illness data in systematic reviews, with the aim to increase generalizability of findings to current and future years [19]. Duplicates between the epidemiology search and costs search were only counted once under the more appropriate search category.

### Study selection, data extraction, and quality assessment

Two reviewers independently screened titles and abstracts and assessed the full reports of potentially eligible studies, along with the dengue research and surveillance databases. A PRISMA flow diagram was used to detail the process of inclusion and exclusion. Using a structured data extraction form, two reviewers individually extracted data from the included reports and subsequently compared notes. Extracted data from journal publications were summarized in a table of characteristics of included studies. The extracted parameters included seroprevalence, serotype distribution, incidence, hospitalization, mortality, direct and indirect costs and expansion factors (EFs), the latter of which are used to account for underreporting of dengue cases when determining cost estimates. EF parameters are detailed in the **S14 Table**. The extracted parameters from dengue research and surveillance databases included incidence rates (IRs), serotype distribution, hospitalization rates and case fatality rates (CFRs).

For published studies, the methodological quality of each selected article was appraised separately by two reviewers using well-accepted quality assessment tools (**S4 and S5 Tables**): (1) for epidemiological studies, the original (cohort/case-control) and adapted (cross-sectional) Newcastle-Ottawa Scales [20, 21]; and (2) for economic studies, a cost-of-illness evaluation checklist adapted from Larg and Moss (2011) [22]. Respectively, the cut-offs for determining poor, fair, and good quality were ≤3, 4–6, and ≥7 for cohort/case-control studies; ≤2, 3–5, and ≥6 for cross-sectional studies; and <50%, 50–<80%, and ≥80% for economic studies.

### Data synthesis

We performed a narrative synthesis and grouped the included studies based on reported outcomes: (1) each of the epidemiologic outcomes and (2) costs of illness. Epidemiological studies were further grouped according to stratified analyses by locality, age or age group, sex, disease severity, serotype, and infection type (i.e., past, primary, secondary, post-secondary). The literature search was not initially intended or designed to be conducted according to these sub-groups; however, the findings are presented in this manner to facilitate robust interpretation. Findings were to be reported separately for studies published in peer-reviewed journals and for gray literature (i.e., not produced by commercial publishers), such as reports retrieved from surveillance databases [16].

Incidence data were summarized using the annual IR per 100,000 person-years. To produce annualized values, we combined weekly or monthly data from the same primary source or computed yearly averages, as appropriate. Imputation was performed to estimate the incidence of dengue by serotype distribution. The IRs were based on midyear population estimates from the Department of Statistics of Singapore [23]. Hospitalization rates referred to the proportion of dengue cases hospitalized per year. CFRs were calculated from the reported number of deaths and cases per year. Other outcomes were summarized using metrics as reported in individual studies. All costs identified were standardized by conversion to 2021 Singapore Dollar (SGD) equivalent values inflated using the average Singapore consumer price index [24].

To present epidemiologic trends from the same primary source, we utilized bar, line, or scatter plots. When presenting findings from multiple primary sources, we used tables to summarize content. We used local polynomial regression fitting to produce the line graphs and Fisher’s exact test or *z*-test to produce 95% confidence intervals (CIs) for CFRs. Statistical analyses were conducted in R 4.3.0 [25].

## Results

### Results of the search

**Fig 1:**
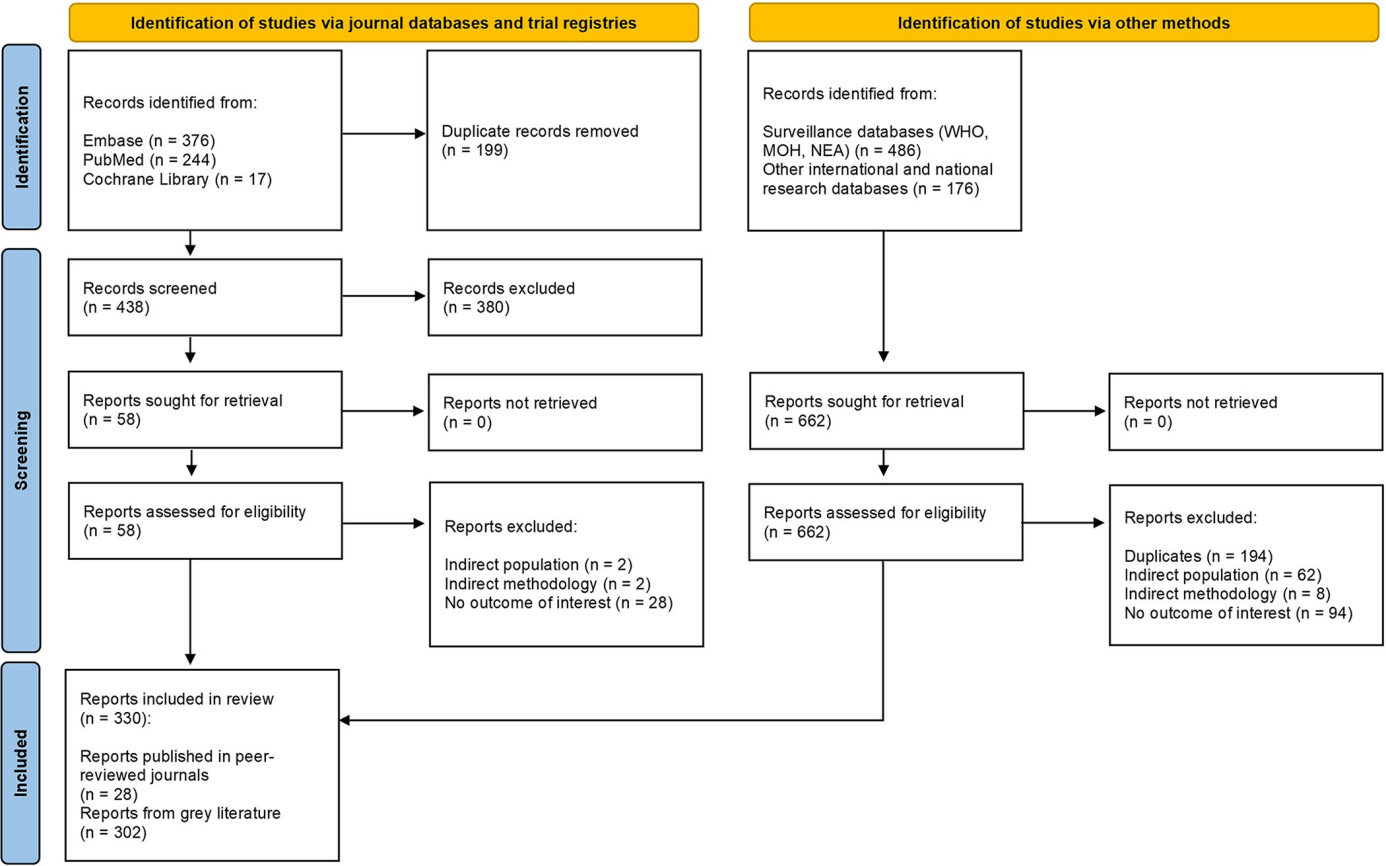
PRISMA flow diagram for epidemiology search. MOH, Ministry of Health; NEA, National Environment Agency; WHO, World Health Organization.

**Fig 2:**
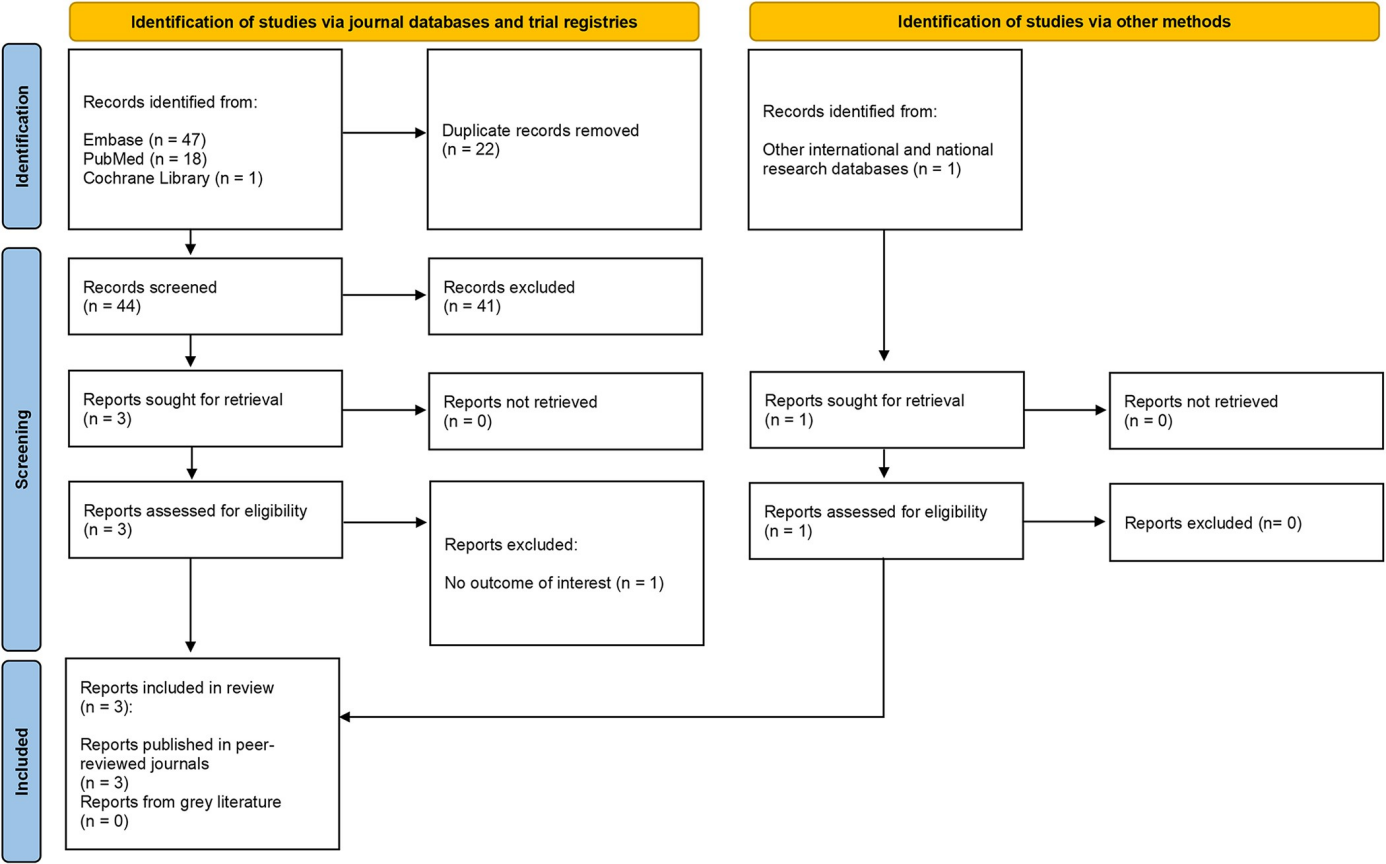
PRISMA flow diagram for costs search.

Our search (**Figs 1 and 2**) yielded 1,366 records from electronic journal databases (703 records) and international/national databases (663 records). After removal of duplicates and screening of titles/abstracts, 61 published reports and all 663 gray literature reports underwent full-text screening. Finally, a total of 333 reports (31 published, 302 gray literature) were included in this review. Out of the 31 included published reports, 28 originated from the epidemiology search while three were acquired from the costs search [12,14,26–54]. All 302 reports included from gray literature were generated by the epidemiology search [55–57].

The published studies were conducted mostly among adults in a hospital or clinic setting. Most (n = 18) published epidemiological studies had a cross-sectional or retrospective cohort design (**Table 1**), whereas two out of three economic studies were cost-effectiveness analyses (CEAs) (**Table 2**). Seroprevalence was the most frequently reported outcome among the published epidemiological studies. Our co-primary outcomes of interest, i.e., dengue incidence and dengue-related costs, were reported in eight of the published epidemiological studies and in all three published economic studies, respectively. None of the included studies reported dengue complications and lengths of treatment, illness, or disability. The gray literature included in this review consisted of publicly available surveillance data from the MOH (n = 282) and NEA (n = 20).

**Table 1:** Characteristics and quality of included publications: Epidemiological studies.

Study identifier	Study design	Data collection period	Population (sample size)^a^^,^^b^	Outcome of interest	Quality rating^c^^,^^d^
Ang 2015a [26]	Cross-sectional	17 March 2010–13 June 2010	Residents aged 18–79 years with residual sera from a national health survey (N = 3,293)	Seroprevalence	Good
Ang 2015b [27]	Cross-sectional	August 2008–July 2010	Inpatients (hospitalization or day surgery) aged 1–17 years without current dengue and with residual sera from a national survey (N = 1,200)	Seroprevalence	Good
Ang 2019 [28]	Retrospective cohort	2003–2017	Hospitalized cases of dengue reported to MOH (N/A)	Incidence, hospitalization, mortality	Good
Arima 2013 [29]	Retrospective cohort	2007–2011	Cases of dengue reported to MOH (N/A)	Incidence, mortality, serotype distribution	Good
Chow 2005 [30]	Cross-sectional	1998–2000	Healthy university graduates aged 19–26 years (N = 184)	Seroprevalence	Fair
Hapuarachchi 2016 [31]	Retrospective cohort	2013–2014	Cases of dengue reported to MOH (N/A)	Incidence, serotype distribution	Good
Koh 2008 [32]	Retrospective cohort	01 January 2005–31 December 2005	Cases of dengue reported to MOH (N/A)	Incidence, mortality, serotype distribution	Good
Leo 2012 [33]	RCT	April 2009–October 2009	Healthy individuals aged 2–45 years without current dengue (N = 585)	Seroprevalence	Good
Ler 2011 [34]	Mixed methods: retrospective cohort, cross-sectional	Retrospective cohort: 2005 and 2007; cross-sectional: 2007	Retrospective cohort: cases of dengue reported to MOH (N/A); cross-sectional: volunteer residents of unspecified age from largest clusters of infection (N = 1,044)	Incidence, mortality, serotype distribution	Good
Low 2015 [35]	Cross-sectional	December 2009–February 2010	Healthy blood donors aged 16–60 years (N = 3,627)	Seroprevalence	Good
Lu 2022 [36]	Prospective cohort	NR	Community-dwelling older adults aged ≥55 years with or without current dengue (N = 844)	Seroprevalence	Good
Lye 2010 [37]	Retrospective cohort	2004	Hospitalized patients aged 17–76 years with dengue (N = 1,971)	Hospitalization, mortality, seroprevalence	Good
Ong 2007 [38]	Case-control	01 January 2004–31 September 2004	Hospitalized adult patients (mean age: 24 years in controls, 47 years in cases) with dengue (N = 42)	Mortality	Good
Park 2020 [39]	RCT (follow-up study)	July 2016–February 2017	Participants aged 9–45 years at first vaccination and completing three vaccinations (N = 585)	Seroprevalence	Good
Pinto 2011 [40]	Retrospective cohort	January 2000–December 2007	Cases of dengue reported to MOH (N/A)	Incidence	Good
Rajarethinam 2018 [41]	Retrospective cohort	2004–2016	Cases of dengue reported to MOH (N/A)	Incidence, mortality	Good
Rouers 2021 [42]	Prospective cohort	NR	Adult patients (mean age: 36.3 years) with suspected dengue and fever <6 days recruited at a hospital (N = 68)	Seroprevalence, hospitalization, serotype distribution	Good
Sadarangani 2016 [43]	Retrospective cohort	2015	Hospitalized patients aged 21–79 years with confirmed dengue (N = 14)	Serotype distribution	Good
Seet 2005 [44]	Mixed methods: prospective cohort, cross-sectional	March 2002–April 2002	Adult Chinese migrant workers (mean age: 33 years) living within a construction site (cross-sectional; N = 274)	Seroprevalence	Fair
Tan 2019 [46]	Cross-sectional	December 2013–February 2014; June–August 2017	Healthy blood donors aged 16–74 years (N = 3,813 in 2013; N = 4,002 in 2017)	Seroprevalence	Good
Thein 2012 [58]	Prospective cohort	2005–2011	Febrile patients aged ≥18 years from clinics (N = 345)	Serotype distribution	Fair
Tricou 2020 [48]	RCT	June 2015–September 2017	Healthy participants aged 21–45 years without current dengue recruited from hospitals (N = 347)	Seroprevalence	Good
Wilder-Smith 2004 [49]	Cross-sectional	August 2002	Asymptomatic volunteering staff and visitors to a hospital, aged 18–45 years (N = 298)	Seroprevalence	Good
Wilder-Smith 2005 [50]	Cross-sectional	August 2002	Healthy staff and visitors to a hospital, aged 18–30 years (N = 164)	Seroprevalence	Good
Yap 2013 [51]	Cross-sectional	2007	Residents of dengue outbreak area, aged 7–85 years (N = 3,939)	Seroprevalence	Good
Yew 2009 [52]	Cross-sectional	September 2004–December 2004	Residents aged 18–74 years with residual sera, from a national health survey (N = 4,152)	Seroprevalence	Good
Yung 2015 [53]	Prospective cohort	April 2005–December 2011	Febrile patients aged 18–87 years from clinics (N = 469)	Serotype distribution	Good
Yung 2016 [54]	Nested case-control in prospective cohort study	April 2005–February 2021	Febrile patients aged ≥18 years from clinics and a hospital (N = 1,703)	Incidence	Good

MOH, Ministry of Health; N/A, not applicable; NR, not reported; RCT, randomized controlled trial.

^a^Sample size with usable data for this review.

^b^Sample size not applicable to MOH-notified cases, which represent outcome groups, not base populations.

^c^Assessed using original and revised Newcastle-Ottawa Scales [20, 21] for epidemiological studies and cost-of-illness evaluation checklist from Larg and Moss (2011) [22] for economic studies.

^d^Thresholds for poor, fair, or good rating: ≤3, 4–6, ≥7 for cohort and case-control studies; ≤2, 3–5, ≥6 for cross-sectional studies; <50%, 50–<80%, ≥80% for economic studies.

**Table 2:** Characteristics and quality of included publications: Economic studies.

Study identifier	Study design	Data collection period	Population (sample size)^a^^,^^b^	Outcome of interest	Quality rating^c^^,^^d^
Carrasco 2011 [14]	Cost-effectiveness analysis	2000–2009	N/A	Direct and indirect costs, expansion factor	Good
Shepard 2013 [12]	Cost-of-illness	2001–2010	N/A	Direct and indirect costs, expansion factor	Good
Soh 2021 [45]	Cost-effectiveness analysis	2010–2020	N/A	Direct and indirect costs, expansion factor	Good

N/A, not applicable.

^a^Sample size with usable data for this review.

^b^Sample size not applicable to MOH-notified cases, which represent outcome groups, not base populations.

^c^Assessed using original and revised Newcastle-Ottawa Scales [20, 21] for epidemiological studies and cost-of-illness evaluation checklist from Larg and Moss (2011) [22] for economic studies.

^d^Thresholds for poor, fair, or good rating: ≤3, 4–6, ≥7 for cohort and case-control studies; ≤2, 3–5, ≥6 for cross-sectional studies; <50%, 50–<80%, ≥80% for economic studies.

In terms of methodological quality (**Table 1**), the majority of published epidemiological studies (25/28 = 89%) received the highest rating (‘good’). The remaining studies were of fair quality, most commonly owing to lack of comparability between outcome groups or non-reporting of the degree of uncertainty (e.g., no CIs). All three publications that were included from the costs search were of good quality (**Table 2**).

### Incidence

**Fig 3:**
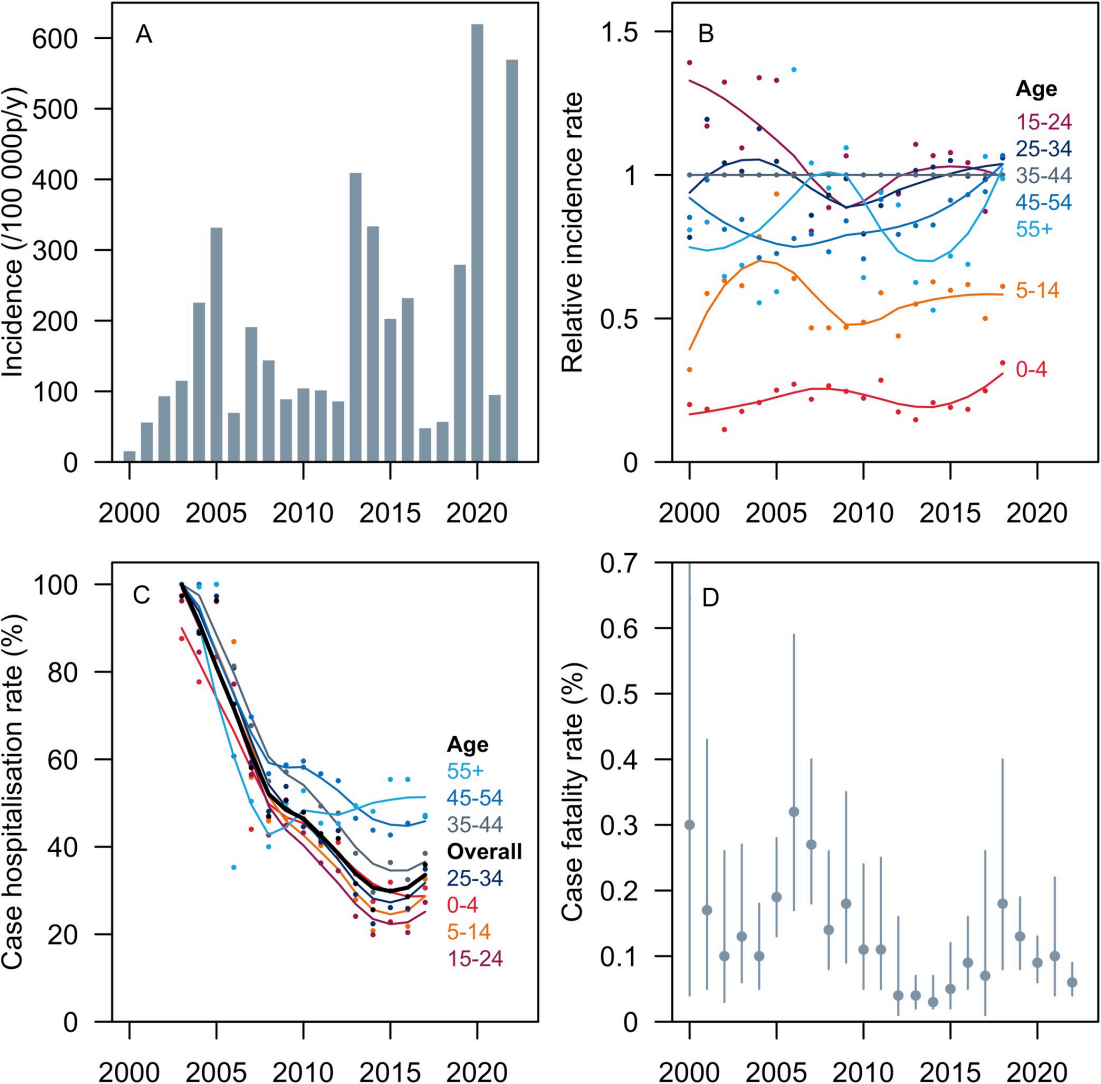
Incidence and severity of dengue in Singapore, 2000 onwards. Panel A, Incidence rate of dengue from 2000 to 2022. Panel B, Relative incidence rate of dengue per age group from 2000 to 2018. Reference group: 35–44 years. Panel C, Hospitalization rate, or proportion of dengue cases hospitalized per year, overall and stratified by age group from 2003 to 2017. Panel D, Dengue case fatality rate from 2000 to 2022 with 95% confidence intervals. Data for panels A and B were retrieved from Singapore Ministry of Health [55, 56]; panel C, from Ang 2019 [28]; panel D, from the Singapore Ministry of Health and National Environment Agency [55–57]. p/y, person-years.

From 2000 to 2021, the MOH reported the lowest dengue IR in 2000 (16.7 cases per 100,000 person-years) and the highest in 2020 (621.1 cases per 100,000 person-years), coinciding with the COVID-19 pandemic [59]. Epidemic patterns, each lasting approximately 2 years, were observed in 2004–2005, 2007–2008, 2013–2014, 2015–2016, and 2019–2020. In 2022, Singapore experienced another dengue outbreak (IR of 570.8 cases per 100,000 person-years) (**Fig 3A, S6 Table**).

MOH surveillance data were secondarily reported in multiple published studies, approximating the findings in this review [28,29,31,40,41].

### Subgroup analysis: by disease severity

Subgroup analysis of MOH surveillance data by disease severity (**S6 Table**) revealed IRs of 0.1–9.2 cases per 100,000 person-years for DHF and 16.5–620.2 cases per 100,000 person-years for dengue fever. Among cases, the risk of DHF ranged from 0.1% to 2.8% per year. It was highest in 2005, coinciding with the peak of Singapore’s first large dengue epidemic in the 21^st^ century, then remained above 2% until 2007. After 2009 (i.e., the end of Singapore’s second epidemic in the 21^st^ century), the risk of DHF declined to and stayed at <1%.

### Subgroup analysis: by age group

Based on MOH surveillance data, the highest IRs were found in the following age groups: 15–24, 25–34, and 35–44 years (**Fig 3B and S7 Table**). Meanwhile, the 0–4 and 5–14 age groups generally had the lowest IRs. Three published studies secondarily reported age-stratified IRs from MOH and found these similar trends [32,34,41].

### Subgroup analysis: by serotype

**Fig 4:**
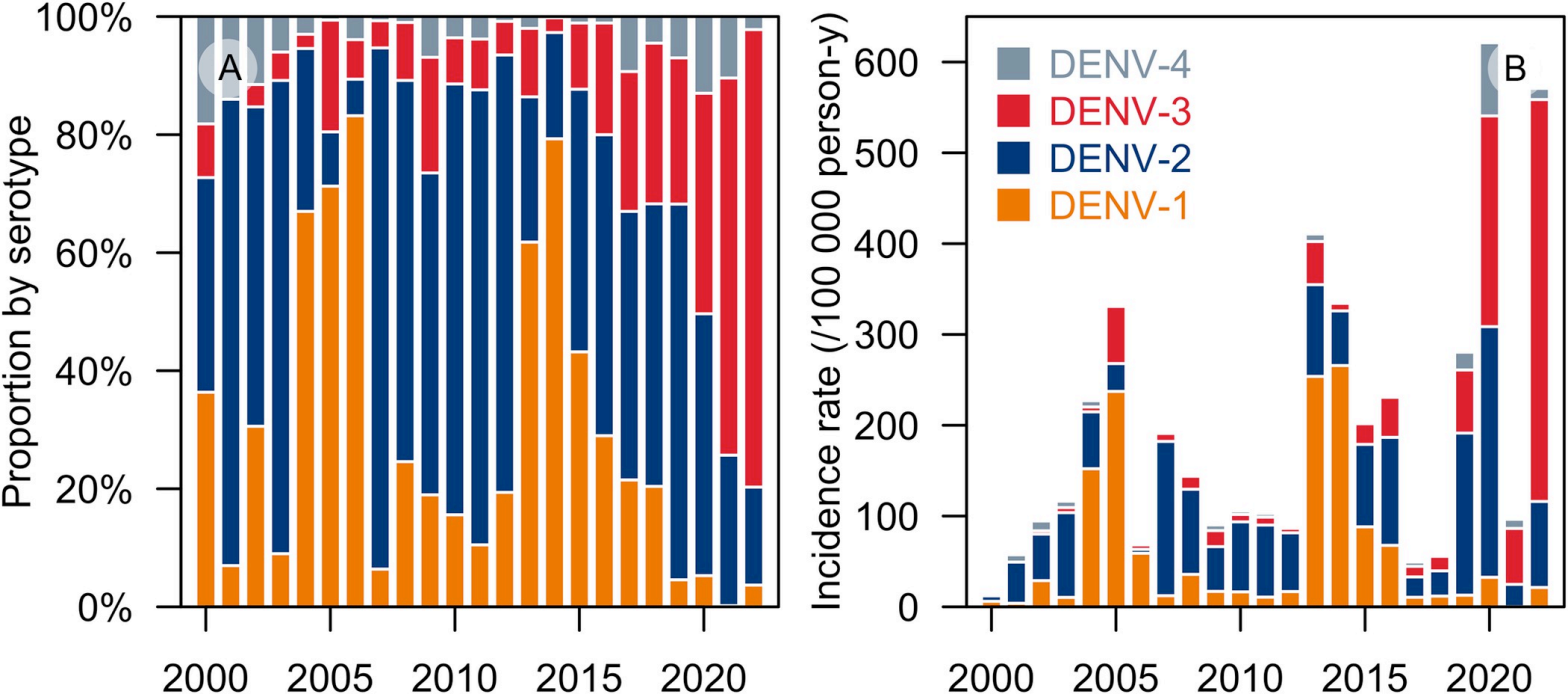
Distribution and incidence of the four dengue virus serotypes in Singapore, 2000 to 2022. Panel A, Proportion of all serotyped cases by serotype and year. Panel B, Incidence rate for each serotype. Data were retrieved from the Singapore Ministry of Health and National Environment Agency [55–57]. DENV, dengue virus; person-y, person-years.

Although the MOH did not present IRs stratified according to serotype, it reported the distribution of the four dengue serotypes from 2000 to 2022 (**Fig 4A and S8 Table**). DENV-2 was the predominant circulating serotype in most of the 22-year period (2000–2003, 2007–2012, 2015–2020) while DENV-1 was predominant in the rest (2004–2006, 2013–2014). DENV-3 only gained prominence in 2021 and remained to be the predominant serotype in 2022, with an average distribution of 77.5%.

These findings were echoed by three published studies secondarily reporting MOH data on serotype distribution [29,31,32]. Five other publications, which were mostly cohort studies in clinics and hospitals, reported serotype distributions consistent with MOH data (**S9 Table**) [34,42,43,53,58].

Using the percentage distribution from MOH to stratify dengue IRs by serotype, we found that for most of 2000–2022, DENV-1 and DENV-2 alternated as the serotype with highest incidence (**Fig 4B and S8 Table**). However, DENV-3 IRs have been rising for the past 6–7 years, recently exceeding those of DENV-1 and -2. DENV-1 incidence, in particular, has dwindled while DENV-4 incidence increased.

### Subgroup analysis: by locality

**Fig 5:**
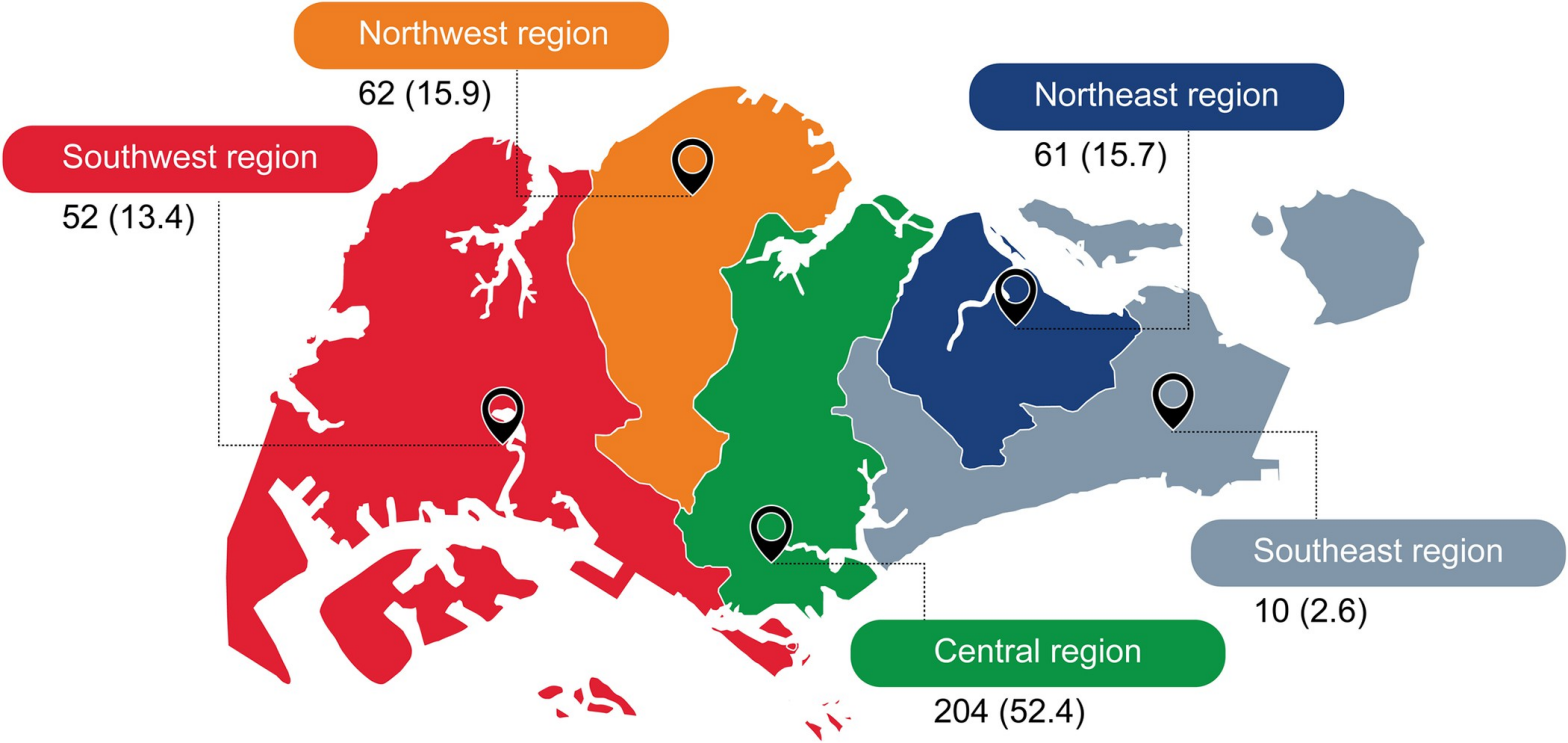
Number of dengue cases by region in Singapore between 2005 and 2013. Data are presented as n (%) of total cases in Singapore (n = 395), using data retrieved from Yung 2009 [54].

A nested case-control study by Yung et al. (2016) [54] in five regions of Singapore between 2005 and 2013 showed that the number of dengue cases varied by region. Respectively, the number of cases was highest and lowest in the Central (204 cases) and Southeast (10 cases) regions (**Fig 5)**. However, the authors found that the Northwest region, which lies near the city of Johor Bahru in Malaysia, had significantly lower odds of dengue on multivariate logistic regression analysis [54].

### Hospitalization

Although the MOH did not routinely report hospitalization rates, a 2018 surveillance report indicated that 78–85% of yearly reported cases from 2000 to 2002 had been hospitalized or had received inpatient treatment [56].

Meanwhile, according to a retrospective cohort study of notified cases from 2003 to 2017, the highest dengue hospitalization rate in Singapore was 92.6%, representing the 2-year average rate in the 2004–2005 epidemic (**Fig 3C and S10 Table**). Subsequently, owing to modifications of hospital admission protocols, hospitalization rates progressively declined to as low as 25.6% in 2014. The mean length of hospitalization among dengue cases over the study period was 3.8 days (range of 1–121 days). Elderly patients (>65 years old) showed significantly longer hospital stays, with an annual mean length of stay of 5–8 days, indicating more severe disease within this age group than others [28].

### Subgroup analysis: by age group

In the same study, a subgroup analysis revealed that the decline in hospitalization rates occurred across all age groups, yet the risk of hospitalization was higher among older adults (≥45 age groups) than among younger patients (**Fig 3C and S10 Table**). Furthermore, the length of hospitalization was significantly longer in the ≥65 age group (mean of 5–8 days) than in other age groups [28].

In contrast, a 2004 retrospective cohort study of hospitalized dengue patients in Tan Tock Seng Hospital found no significant difference between patients aged <60 years and those aged ≥60 years in terms of hospitalization duration (median of 4 days in both groups) [37].

### Subgroup analysis: by infection type

Subgroup analysis of hospitalization by infection type was also available from a prospective cohort study of dengue suspects, which illustrated that the risk of hospitalization was higher with secondary infection than with primary infection (50% vs 20%) [42].

### Mortality

Since 2002, the annual CFRs for dengue and their corresponding 95% CI upper bounds have all been less than 1% (**Fig 3D and S11 Table**). More specifically, in the last 10 years (2010–2022), the CFRs and upper bounds have not exceeded 0.5%. As can be expected, the CIs were narrowest during epidemic years, when the number of cases was larger. Conversely, the wide CIs reflected the uncertainty of estimates for non-epidemic years, even if some CFRs were artefactually high.

Similar CFR findings were reported in five published studies, which also used secondary data from MOH surveillance [28,29,32,34,41].

### Subgroup analysis: by disease severity

Mortality data stratified by disease severity (at presentation) were available from MOH surveillance until 2011 (**S12 Table**), after which the MOH stopped reporting stratified data, possibly due to the 2009 changes in WHO case definitions [2]. As expected, CFRs for dengue fever (0.1–0.3%) were generally lower than those for DHF, which had a peak of 10.9% in 2009. Three published studies also reported CFRs for DHF within these ranges. Koh et al. (2008) [32] and Ler et al. (2011) [34] secondarily reported MOH surveillance data, while Ong et al. (2007) [38] conducted a 2004 case-control study of patients in Tan Tock Seng Hospital.

### Subgroup analysis: by age group

The MOH did not report age-stratified deaths, but Lye et al. (2010) [37] found no statistically significant difference between the ≥60 and <60 age groups in terms of CFRs (0.0 vs 0.1%).

### Seroprevalence

Data on seroprevalence were not routinely part of MOH surveillance. Nevertheless, 16 published studies reported seroprevalence data from populations of varied age, health status, and healthcare setting (**S13 Table**). Across these studies, the most common study design was cross-sectional, while the most common method of testing for antibodies was enzyme-linked immunosorbent assay (ELISA). Collectively, a wide range of seroprevalence (10.0–73.7%) was reported [26,27,30,33,35–37,39,42,44,46,48–52].

**Fig 6:**
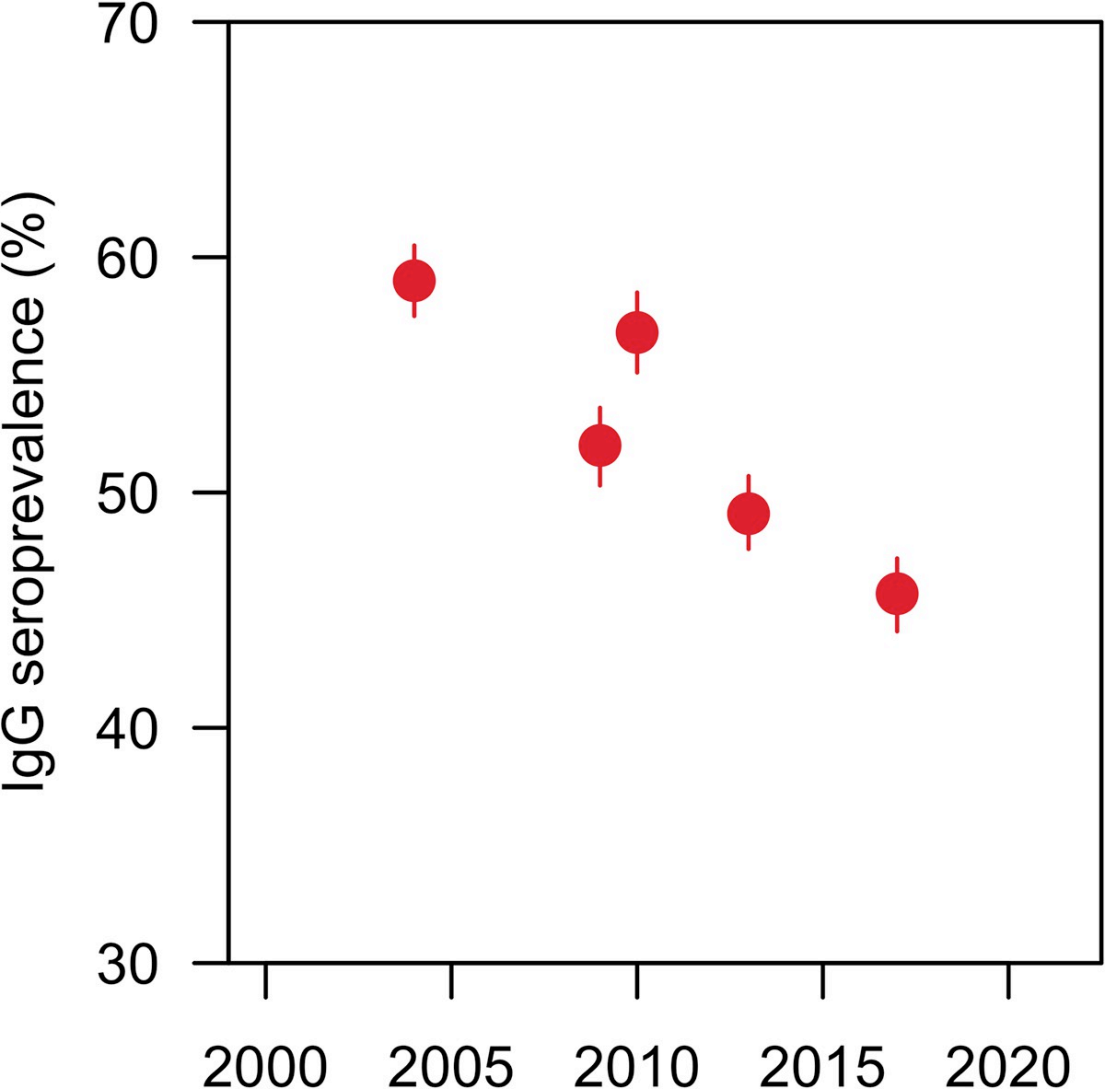
Crude seroprevalence of dengue IgG during select years in Singapore. Lines represent 95% confidence intervals. Age ranges in years were 18–74 (2004), 16–60 (2009), 18–79 (2010), and 16–74 (2013 and 2017), using data retrieved from Yew 2009 [52], Low 2015 [35], Ang 2015a [26], and Tan 2019 [46], respectively. IgG, immunoglobulin G.

Four of these studies reported the results of nationwide serosurveys that determined the presence of dengue immunoglobulin G (IgG) to detect past infection (**Fig 6**). Low et al. (2015) [35] and Tan et al. (2019) [46] involved blood donors; Yew et al. (2009) [52] and Ang et al. (2015) [26] conducted national health surveys. The studies (N = 3,293–4,152) demonstrated a generally decreasing trend in seroprevalence, from 59% in 2004 to 45.7% in 2017 [26,35,46,52].

Meanwhile, in two studies, IgG seroprevalence (35.0–55.9%) was determined upon hospital admission among patients with current suspected or confirmed dengue, representing past dengue infection [37,42]. Two other studies determined the seroprevalence of dengue immunoglobulin M (IgM) to detect recent infection. In a 2007 study conducted among residents of dengue outbreak areas, seroprevalence of IgM or IgG was high (65.9%), as was the prevalence of inapparent dengue (78%) among those who were IgM-positive [51]. Contrarily, a study among Chinese migrant workers living within a construction site during the 2002 outbreak showed a low IgM seroprevalence (10%) [44].

### Subgroup analysis: by age group

Multiple published studies reported age-stratified seroprevalence (**S13 Table**). However, age groupings in the four national serosurveys [21,31,43,49] were inconsistent while four other, more localized studies [27,33,37,49] had varying study populations and methodologies. Despite this heterogeneity, seroprevalence generally increased with age: ~10–30% in children aged <18 years and ~70–90% in older adults aged ≥55 years (as measured by ELISA) [26,27,33,35,46,52]. Substantial falls in age-specific seroprevalence have occurred over the first two decades of the 21^st^ century [46].

### Subgroup analysis: by serotype

Subgroup analyses of seroprevalence by serotype were available from four published studies of varying cohort composition and study design (**S13 Table**) [33,35,48,50]. The largest of these studies [35] showed a higher seroprevalence of antibodies against DENV-1 and -2 versus the other serotypes in 2009–2010, corresponding with the contemporaneously circulating dengue serotypes. Furthermore, as the age of subjects increased, the studies found a higher seroprevalence of antibodies against each serotype and a higher frequency of a multi-serotype pattern [33,35].

### Economic burden

The three published economic studies varied in terms of component cost estimates, sources, and assumptions (**Tables 3 and S14**). Albeit not directly stated, a societal perspective was adopted in all three studies, based on their consideration of both costs borne by the patient (or family) and costs borne by the government as a payor [12,14,45].

**Table 3:** Characteristics and results of published studies reporting the total costs of illness from dengue in Singapore.

Study identifier	Period of estimation	Method	Mean aggregated cost to Singapore per year, million 2021 SGD (95% CI)		
			Direct	Indirect	Total
Carrasco 2011 [14]	2000–2009	*Human capital, constant symptomatic rates	–	–	68 (54,95)
		*Human capital, age-dependent symptomatic rate	–	–	107 (87,131)
		Friction cost, constant symptomatic rate	–	–	58 (40,82)
		Friction cost, age-dependent symptomatic rate	–	–	92 (74,119)
Shepard 2013 [12]	2001–2010	^#^Human capital	41 (23,64)	69 (44,93)	110 (69,155)
Soh 2021 [45]	2010–2020	Friction cost, constant symptomatic rate	–	–	148

^#^The human capital method values lost time or premature death using an individual’s gross earnings, *calculated from gross domestic product per capita and the total years of premature life lost based on the discounted, weighted life expectancy using WHO life tables for each country. The friction cost method assumes job absenteeism or death lead to productivity losses, which in both studies is assumed to be 30 days for fatalities and to last as long as symptoms in non-fatal cases, and productivity losses are offset according to the elasticity of annual labor time versus labor productivity [12, 14, 45]. Costs originally published in 2010 USD were converted to 2010 SGD and standardized to 2021 values using the ratio of 2021 to 2010 average consumer price indices; costs were rounded to the nearest million. CI, confidence interval;–, not reported.

Carrasco et al. (2011) [14] estimated the costs of dengue for 2000–2009 as part of a CEA of a potential dengue vaccination program. The study applied four approach permutations using a combination of two sets of methods: (1) human-capital versus friction-cost method and (2) constant versus age-dependent symptomatic rate. Among these approaches, the most conservative cost estimates were produced via the friction-cost method and a constant symptomatic rate. Direct costs (including costs of hospitalization or visits by ambulatory patients, in addition to transport costs) constituted a higher proportion of the total costs than indirect costs (such as average household service losses, cost of providing schooling, cost of absence from work; **S14 Table**); such costs of productivity loss (indirect costs) comprised 21–29% of the total costs, depending on the approach. Additionally, the authors reported estimates with and without the costs of Singapore’s contemporary vector control program (i.e., habitat reduction, insecticide spraying), which corresponded to SGD 68 million per year, according to their consultations with the NEA [14].

Meanwhile, Shepard et al. (2013) [12] performed a cost-of-illness study among countries in Southeast Asia, including Singapore. Using a human capital approach and the component costs listed by Carrasco et al. (2011) [14], the study produced probabilistic estimates of the direct and indirect costs per case of hospitalized and ambulatory dengue for 2001–2010 (**S14 Table**). Compared with the study by Carrasco et al. (2011) [14], higher annual total costs were reported. Furthermore, indirect costs constituted a higher proportion of the total costs than direct costs (63% vs 37%) [12].

Lastly, Soh et al. (2021) [45] undertook a CEA of *Wolbachia* interventions, whereby the authors estimated dengue-related costs via the four approaches adopted by Carrasco et al. (2011) [14]. Using the same, most conservative method, the study found that the average annual total costs for 2010–2020 were twice as high as those reported by Carrasco et al. (2011) [14] for the previous decade (2000–2009) [45].

Expansion factors (EFs) affected cost estimates in the economic studies. EF values were mostly similar between the studies by Carrasco et al. (2011) [14] and Soh et al. (2021) [45] but different from that by Shepard et al. (2013) [12] (**S14 Table**), possibly explaining the discrepancy between results by Carrasco et al. (2011) [14] and Shepard et al. (2013) [12] despite their analogous reference periods. Carrasco et al. (2011) [14] distinguished EFs along three dimensions: age, symptomatic rate, and healthcare setting. EFs were higher with increasing age and for the age-dependent (vs constant) symptomatic rate. Generally, EFs for ambulatory cases (1.7–24.2) were also higher than those for hospitalized cases (1.4–3.4). Soh et al. (2021) [45] adapted these EFs from the methodology of Carrasco et al. (2011) [14]. However, Soh et al. (2021) [45] deviated by using an EF of 1 for hospitalized cases, based on the assumption that all suspected cases were being tested prior to hospital admission. In contrast, Shepard et al. (2013) [12] applied EFs to non-fatal cases, aside from fatal cases. Moreover, the authors divided EFs into three types without stratification by age: hospitalized, ambulatory, and overall. Consistent with the study by Carrasco et al. (2011) [14], ambulatory EF (5.0) was higher than hospitalized EF (2.5) [12,14,45].

## Discussion

MOH surveillance was the source of most epidemiologic data shown in this review. In terms of epidemiologic burden, Singapore experienced five large epidemics in the past 20 years, interspersed with periods of endemic dengue [9], and reached peak IR during the latest outbreak in 2020 amidst the COVID-19 pandemic [59]. Our subgroup analyses revealed that dengue IRs were highest for the serotypes DENV-2 (previously) and DENV-3 (currently) and in the 15–44 age group. For the past 10 years, the risk of DHF has been low (<1%). In terms of economic burden, the included studies demonstrated higher dengue-related costs in the past decade than in the immediately preceding decade (2000–2009), corresponding with an increased dengue burden compared with the preceding decade [45]. Indirect costs of dengue contributed greatly to high total costs. Additionally, we found that in recent years, hospitalization occurred in about one-third of cases and more frequently among adults aged ≥45 years. Nevertheless, mortality from dengue has been consistently low (CFR <1%) for the last 20 years. Lastly, the most recent seroprevalence data illustrated that dengue IgG, although increasing with age, was present in <50% of the general population and has been falling over time.

Based on this review, research gaps remain in Singapore in terms of stratified epidemiologic data, such as incidence by age group, which may be added to routine MOH surveillance and reporting to develop targeted interventions for dengue control. As with the study by Ang et al. (2019) [28], national hospital-claims databases could be used to acquire hospitalization rates and CFRs according to disease severity, especially if out-of-hospital deaths are assumed to be absent. Meanwhile, seroprevalence according to serotype could be obtained through incorporation into the design of national serosurveys, as done by Low et al. (2015) [35], or the use of residual sera, as done by Ang et al. (2015) [26,27], although the cost of doing so may be high.

CFRs stratified by infection type (primary, secondary, post-secondary) are also currently missing from the local literature. The risk of severe dengue may be higher in secondary versus primary infection owing to antibody-dependent enhancement, whereby viral replication is increased from priming with pre-existing antibodies to one serotype [60,61]. Alternatively, the risk of severe disease in post-secondary infection may be no different, or even lower, than in primary or secondary infection [62,63]. To further understand these nuances, more prospective cohort studies such as that by Rouers et al. (2021) [42] are warranted. In addition, evaluating the impact of interventions for dengue control will require updating of some epidemiologic data, including dengue hospitalization rates, length of hospitalization, and national seroprevalence. Relatedly, EFs in the identified economic studies were estimated using data that are now >10 years old. Hence, studies that estimate EFs are essential, as performed by Tan et al. (2019) [46], who estimated an EF of 6 for 2014–2017.

Akin with epidemiologic data, stratification of costs is presently lacking in local literature (e.g., by age group, by epidemic versus non-epidemic year, by societal or governmental perspective). For instance, the mixed cost estimates by Soh et al. (2021) [45] were based on an assortment of costs from public hospitals and private outpatient facilities, as well as subsidized and out-of-pocket costs. Claims databases, surveys of clinics and hospitals, and line-item costing are some possible sources of more stratified data. Proper costing would also involve all agencies, institutions, and personnel in the costs [64]. Furthermore, the macroeconomic burden of dengue in Singapore, including its impact on tourism and other public sectors (e.g., education, trade, domestic services), remains to be elucidated as it has been in Brazil, where dengue is similarly endemic and the macroeconomic impact is considerable [65]. While the existing cost-of-illness studies have discussed the microeconomic impact of dengue in Singapore in terms of average household services lost, average productivity loss per absent day at work, DALY’s, etc. [12,14,45], less adequately described in the literature is the economic impact of dengue on household consumption potential of the affected patient owing to an increase in healthcare expenditure.

The available evidence has some limitations, such as those concerning MOH surveillance data. Underreporting remains a possibility in passive surveillance. Patients with inapparent or mild dengue infection may not seek care amidst their potential for viral transmission [64], and health-seeking behavior may change over time, such as during the COVID-19 pandemic. Merging multiple data sources (surveillance, cohort studies, community-based surveys) could address this issue [64]. Nevertheless, some evidence points to improving dengue reporting practices in Singapore. The reduction in dengue seroprevalence despite an increasing incidence suggests an “unmasking” of infections from better reporting [46], rather than a genuine increase in incident infections. The decline in risk of DHF may also be explained by higher ascertainment rates of milder dengue disease, which align with improving case notification in Singapore [46].

Data from published epidemiologic literature were of good quality but similarly limited. We found scarce primary data while most secondary data were aggregated. Without the benefit of individual patient data, the capacity for subgroup analysis or synthesis is restricted. Additionally, only a few prospective studies were identified.

A number of contextual features are distinct to Singapore. The enduring vulnerability of Singapore to dengue epidemics is potentially a result of low and falling herd immunity and persistent viral transmission [66]. In turn, low herd immunity is caused by historic falls in the force of infection, which had begun to decline in Singapore in the 1990s [46]. Presently, Singapore has the lowest force of infection among endemic countries in Asia [67]. Some have attributed this phenomenon to its aging population, as more older individuals represent more immunity against dengue, but it is an unlikely primary cause in light of decreasing seroprevalence in Singapore [46]. The more probable reason is Singapore’s vector control program, which has effectively lowered transmission risk [9,46,67]. Paradoxically, the resulting low herd immunity decreases the effectiveness of vector control programs, because the risk of infection is higher even if the number of vectors is lower [46]. Notwithstanding vector control, persistent transmission of all dengue serotypes occurs in Singapore, whereas across-type interactions contribute to the emergence of outbreaks [68]. Coupled with climate change, increased travel to and from Singapore, as measured through air passenger flow, can lead to DENV lineage movement, long-run local transmission, and seeding of outbreaks [9,69,70].

Our review process was limited by the absence of a meta-analysis. The variability in reported outcomes among identified studies, limited number of primary sources, and inconsistent groupings across included studies were factors that inhibited a quantitative synthesis. Moreover, for purposes of summarization, we performed some transformation of data (e.g., averaging, standardization, imputation) that potentially introduced information bias. Another important limitation is that most of the literature predates the deployment of novel technologies for vector control, namely the *Wolbachia* program and the use of mass Gravitrapping for targeted vector control [9], as well as the licensing of the first dengue vaccine [71].

## Conclusion

Singapore, where dengue is endemic, has seen a dramatic rise in dengue incidence rates over the past two decades but, simultaneously, a notably low force of infection, which is likely the result of effective and nationally funded comprehensive dengue control strategies inclusive of active inter-epidemic epidemiological surveillance that is largely unseen elsewhere in the region [9,66,72,73]. However, as for other countries of similar climate where dengue is endemic, there is room for improvement with respect to government-led surveillance and reporting measures [72,73]. In our review of 20 years’ worth of data in Singapore, we identified that despite improved data collection from mandatory passive surveillance, there remains a need for sufficiently detailed and up-to-date data on the epidemiologic and economic burden of dengue in Singapore. Owing to the complexities of dengue in terms of disease transmission and pathophysiology, stratified analyses by age, severity, infection type, and serotype are indispensable, and we would strongly recommend the Ministry of Health to consider implementation of surveillance and reporting measures (e.g., national serosurveys to identify seroprevalence according to serotype) to capture and publish such subgroup data for subsequent analysis. A robust reporting system can ultimately provide the data that can be used to accurately determine the cost-effectiveness of interventions for dengue control and focus research and development efforts accordingly.

## Supporting information

S1 Table Search strategy for ‘epidemiology search’. (DOCX)

S2 Table Search strategy for ‘costs search’. (DOCX)

S3 Table International and national research databases searched. (DOCX)

S4 Table Questions in the quality assessment tool for epidemiological studies. (DOCX)

S5 Table Questions in the quality assessment tool for economic studies. (DOCX)

S6 Table Incidence rate of dengue by severity from 2000 to 2022 in Singapore. (DOCX)

S7 Table Incidence rate of dengue by age group from 2000 to 2018 in Singapore. (DOCX)

S8 Table Incidence rate of dengue by serotype from 2000 to 2022 in Singapore. (DOCX)

S9 Table Characteristics and results of published studies reporting dengue serotype distribution in Singapore. (DOCX)

S10 Table Dengue hospitalization rate by age group from 2003 to 2017 in Singapore. (DOCX)

S11 Table Dengue case fatality rate from 2000 to 2022 in Singapore. (DOCX)

S12 Table Dengue case fatality rate by disease severity for select years in Singapore. (DOCX)

S13 Table Characteristics and results of published studies reporting dengue seroprevalence in Singapore. (DOCX)

S14 Table Characteristics of published studies reporting cost of illness from dengue in Singapore. (DOCX)

S15 Table PRISMA 2020 for abstracts checklist. (DOCX)

S16 Table PRISMA 2020 checklist. (DOCX)
