# Supplementary material for: The epidemiologic and economic burden of dengue in Singapore: A systematic review
Source: PLoS Negl Trop Dis. 2024 Jun 10;18(6):e0012240. doi: 10.1371/journal.pntd.0012240 (PMC11192419; doi:10.1371/journal.pntd.0012240)
Supplement: S1 Table — (DOCX) [file pntd.0012240.s001.docx]

**S1 Table.** Search strategy for ‘epidemiology search’.

| **Search Number** | **Query** | | | **Results** | | |
| --- | --- | --- | --- | --- | --- | --- |
|  | **Embase** | **PubMed** | **Cochrane Library** | **Embase** | **PubMed** | **Cochrane Library** |
| #1 | 'dengue'/de OR 'severe dengue'/de OR 'dengue virus'/de OR 'dengue hemorrhagic fever'/de OR 'dengue shock syndrome'/de OR 'dengue virus 1'/de OR 'dengue virus 2'/de OR 'dengue virus 3'/de OR 'dengue virus 4'/de | dengue[mesh:noexp] OR severe dengue[mesh:noexp] OR Dengue Virus[mesh:noexp] OR E protein TH Sman, Dengue virus[Supplementary Concept] OR E-glycoprotein, Dengue virus type 1[Supplementary Concept] OR NS1 protein, Dengue virus type 2[Supplementary Concept] OR E-glycoprotein, Dengue virus type 2[Supplementary Concept] OR NS2A protein, Dengue virus type 2[Supplementary Concept] OR prM protein, Dengue virus type 3[Supplementary Concept] OR E protein, Dengue virus type 3[Supplementary Concept] OR NS1 protein, Dengue virus type 3[Supplementary Concept] OR E-glycoprotein, Dengue virus type 3[Supplementary Concept] OR glycoprotein E, dengue virus type 4[Supplementary Concept] OR NS1 protein, Dengue virus type 4[Supplementary Concept] | MeSH descriptor: [Dengue] this term only OR MeSH descriptor: [Severe Dengue] this term only OR MeSH descriptor: [Dengue Virus] this term only | 33,255 | 18,319 | 344 |
| #2 | 'dengue':ti,ab,tn,tt,DE,mn,de,kw OR 'severe dengue':ti,ab,tn,tt,DE,mn,de,kw OR 'dengue virus':ti,ab,tn,tt,DE,mn,de,kw OR 'dengue h$emorrhagic fever':ti,ab,tn,tt,DE,mn,de,kw OR 'alarm signs':ti,ab,tn,tt,DE,mn,de,kw | dengue[tw] OR severe dengue[tw] OR dengue virus[tw] OR dengue hemorrhagic fever[tw] OR dengue haemorrhagic fever[tw] OR alarm signs[tw] | (dengue):ti,ab,kw OR ("severe dengue"):ti,ab,kw OR ("dengue virus"):ti,ab,kw OR ("dengue hemorrhagic fever"):ti,ab,kw OR ("dengue haemorrhagic fever"):ti,ab,kw OR (“alarm signs”):ti,ab,kw | 38,415 | 26,934 | 805 |
| #3 | 'epidemiology'/de OR 'incidence'/de OR 'prevalence'/de OR 'epidemiology':ti,ab,tn,tt,DE,mn,de,kw OR 'incidence':ti,ab,tn,tt,DE,mn,de,kw OR 'frequency':ti,ab,tn,tt,DE,mn,de,kw OR 'risk':ti,ab,tn,tt,DE,mn,de,kw OR 'rate':ti,ab,tn,tt,DE,mn,de,kw OR 'proportion':ti,ab,tn,tt,DE,mn,de,kw OR 'cases':ti,ab,tn,tt,DE,mn,de,kw OR 'number':ti,ab,tn,tt,DE,mn,de,kw OR 'report':ti,ab,tn,tt,DE,mn,de,kw | epidemiologic studies[mesh:noexp] OR epidemiology[mesh:noexp] OR incidence[mesh:noexp] OR prevalence[mesh:noexp] OR epidemiology[tw] OR incidence[tw] OR frequency[tw] OR risk[tw] OR rate[tw] OR proportion[tw] OR cases[tw] OR number[tw] OR report[tw] | MeSH descriptor: [Epidemiologic Studies] this term only OR  MeSH descriptor: [Epidemiology] this term only OR MeSH descriptor: [Incidence] this term only OR MeSH descriptor: [Prevalence] this term only OR (epidemiology):ti,ab,kw OR (incidence):ti,ab,kw OR (frequency):ti,ab,kw OR (risk):ti,ab,kw OR (rate):ti,ab,kw OR (proportion):ti,ab,kw OR (cases):ti,ab,kw OR (number):ti,ab,kw OR (report):ti,ab,kw | 16,223,810 | 11,126,264 | 846,497 |
| #4 | 'morbidity'/de OR 'mortality'/de OR 'mortality risk'/de OR 'mortality rate'/de OR 'fatality'/de OR 'death':ti,ab,tn,tt,DE,mn,de,kw OR 'case fatality rat*':ti,ab,tn,tt,DE,mn,de,kw OR 'fatality rat*':ti,ab,tn,tt,DE,mn,de,kw OR 'case fatality risk*':ti,ab,tn,tt,DE,mn,de,kw OR 'severity':ti,ab,tn,tt,DE,mn,de,kw OR 'hospitali*ation rate*':ti,ab,tn,tt,DE,mn,de,kw | morbidity[mesh:noexp] OR mortality[mesh:noexp] OR fatal outcome[mesh:noexp] OR death[tw] OR case fatality rat*[tw] OR fatality rat*[tw] OR case fatality risk*[tw] OR severity[tw] OR hospitalisation rate*[tw] OR hospitalization rate*[tw] | MeSH descriptor: [Morbidity] this term only OR MeSH descriptor: [Mortality] this term only OR MeSH descriptor: [Fatal Outcome] this term only OR (death):ti,ab,kw OR (“case fatality rate”):ti,ab,kw OR (“case fatality rates”):ti,ab,kw OR (“case fatality ratio”):ti,ab,kw OR (“case fatality ratios”):ti,ab,kw OR (“fatality rate”):ti,ab,kw OR (“fatality rates”):ti,ab,kw OR (“case fatality risk”):ti,ab,kw OR (“case fatality risks”):ti,ab,kw OR (severity):ti,ab,kw OR (“hospitalization rate”):ti,ab,kw OR (“hospitalization rates”):ti,ab,kw OR (“hospitalisation rate”):ti,ab,kw OR (“hospitalisation rates”):ti,ab,kw | 3,714,936 | 1,795,100 | 181,278 |
| #5 | 'seroprevalence':ti,ab,tn,tt,DE,mn,de,kw OR 'serotype*':ti,ab,tn,tt,DE,mn,de,kw OR 'serogroup':ti,ab,tn,tt,DE,mn,de,kw OR 'serostatus':ti,ab,tn,tt,DE,mn,de,kw OR 'serotype distribution':ti,ab,tn,tt,DE,mn,de,kw | seroprevalence[tw] OR serotype*[tw] OR serogroup[tw] OR serostatus[tw] OR serotype distribution[tw] | (seroprevalence):ti,ab,kw OR (serotype):ti,ab,kw OR (serotypes):ti,ab,kw OR (serogroup):ti,ab,kw OR (serostatus):ti,ab,kw OR (“serotype distribution”):ti,ab,kw | 122,967 | 93,023 | 3,033 |
| #6 | 'cross protection':ti,ab,tn,tt,DE,mn,de,kw OR 'primary infection':ti,ab,tn,tt,DE,mn,de,kw OR 'secondary infection':ti,ab,tn,tt,DE,mn,de,kw OR 'post-secondary infection':ti,ab,tn,tt,DE,mn,de,kw | cross protection[tw] OR primary infection[tw] OR secondary infection[tw] OR post-secondary infection[tw] | (“cross protection”):ti,ab,kw OR (“primary infection”):ti,ab,kw OR (“secondary infection”):ti,ab,kw OR (“post secondary infection”):ti,ab,kw | 23,524 | 15,383 | 573 |
| #7 | 'seasonal variation'/de OR 'season*':ti,ab,tn,tt,DE,mn,de,kw | seasons[mesh:noexp] OR season*[tw] | MeSH descriptor: [Seasons] this term only OR (“season”):ti,ab,kw OR (“seasons”):ti,ab,kw OR (“seasonal”):ti,ab,kw OR (“seasonality”):ti,ab,kw OR (“seasonally”):ti,ab,kw | 261,434 | 240,543 | 10,644 |
| #8 | singapore:ti,ab,tn,tt,DE,mn,de,kw | Singapore[tw] | (singapore):ti,ab,kw | 33,691 | 22,333 | 1,681 |
| #9 | #1 OR #2 | | | 38,415 | 26,934 | 805 |
| #10 | #3 OR #4 OR #5 OR #6 OR #7 | | | 17,799,234 | 12,120,160 | 912,953 |
| #11 | #9 AND #10 | | | 25,963 | 17,754 | 581 |
| #12 | #8 AND #11 | | | 470 | 317 | 17 |
| #13 | #12, filters: humans, English, 2000–2022 | | | 376 | 244 | 17 |
