# Supplementary material for: The epidemiologic and economic burden of dengue in Singapore: A systematic review
Source: PLoS Negl Trop Dis. 2024 Jun 10;18(6):e0012240. doi: 10.1371/journal.pntd.0012240 (PMC11192419; doi:10.1371/journal.pntd.0012240)
Supplement: S2 Table — (DOCX) [file pntd.0012240.s002.docx]

**S2 Table.** Search strategy for ‘costs search’.

| **Search Number** | **Query** | | | **Results** | | |
| --- | --- | --- | --- | --- | --- | --- |
|  | **Embase** | **PubMed** | **Cochrane Library** | **Embase** | **PubMed** | **Cochrane Library** |
| #1 | 'dengue'/de OR 'severe dengue'/de OR 'dengue virus'/de OR 'dengue hemorrhagic fever'/de OR 'dengue shock syndrome'/de OR 'dengue virus 1'/de OR 'dengue virus 2'/de OR 'dengue virus 3'/de OR 'dengue virus 4'/de | dengue[mesh:noexp] OR severe dengue[mesh:noexp] OR Dengue Virus[mesh:noexp] OR E protein TH Sman, Dengue virus[Supplementary Concept] OR E-glycoprotein, Dengue virus type 1[Supplementary Concept] OR NS1 protein, Dengue virus type 2[Supplementary Concept] OR E-glycoprotein, Dengue virus type 2[Supplementary Concept] OR NS2A protein, Dengue virus type 2[Supplementary Concept] OR prM protein, Dengue virus type 3[Supplementary Concept] OR E protein, Dengue virus type 3[Supplementary Concept] OR NS1 protein, Dengue virus type 3[Supplementary Concept] OR E-glycoprotein, Dengue virus type 3[Supplementary Concept] OR glycoprotein E, dengue virus type 4[Supplementary Concept] OR NS1 protein, Dengue virus type 4[Supplementary Concept] | MeSH descriptor: [Dengue] this term only OR MeSH descriptor: [Severe Dengue] this term only OR MeSH descriptor: [Dengue Virus] this term only | 33,255 | 18,319 | 344 |
| #2 | 'dengue':ti,ab,tn,tt,DE,mn,de,kw OR 'severe dengue':ti,ab,tn,tt,DE,mn,de,kw OR 'dengue virus':ti,ab,tn,tt,DE,mn,de,kw OR 'dengue h$emorrhagic fever':ti,ab,tn,tt,DE,mn,de,kw OR 'alarm signs':ti,ab,tn,tt,DE,mn,de,kw | dengue[tw] OR severe dengue[tw] OR dengue virus[tw] OR dengue hemorrhagic fever[tw] OR dengue haemorrhagic fever[tw] OR alarm signs[tw] | (dengue):ti,ab,kw OR ("severe dengue"):ti,ab,kw OR ("dengue virus"):ti,ab,kw OR ("dengue hemorrhagic fever"):ti,ab,kw OR ("dengue haemorrhagic fever"):ti,ab,kw OR (“alarm signs”):ti,ab,kw | 38,415 | 26,934 | 805 |
| #3 | 'health care cost'/de OR 'cost of illness'/de OR 'hospitalization'/de OR 'hospital cost'/de OR 'economic aspect'/de | health care costs[mesh:noexp] OR cost of illness[mesh:noexp] OR hospitalization[mesh:noexp] OR hospital costs[mesh:noexp] OR economics[mesh:noexp] | MeSH descriptor: [Health Care Costs] this term only OR MeSH descriptor: [Cost of Illness] this term only OR MeSH descriptor: [Hospitalization] this term only OR MeSH descriptor: [Hospital Costs] this term only OR MeSH descriptor: [Economics] this term only | 804,527 | 228,153 | 9,112 |
| #4 | economic aspect':ti,ab,tn,tt,DE,mn,de,kw OR 'financial aspect':ti,ab,tn,tt,DE,mn,de,kw OR 'cost* of care':ti,ab,tn,tt,DE,mn,de,kw OR 'direct cost*':ti,ab,tn,tt,DE,mn,de,kw OR 'drug cost*':ti,ab,tn,tt,DE,mn,de,kw OR 'medical cost*':ti,ab,tn,tt,DE,mn,de,kw OR 'physician cost*':ti,ab,tn,tt,DE,mn,de,kw OR 'nurse cost*':ti,ab,tn,tt,DE,mn,de,kw OR 'inpatient cost*':ti,ab,tn,tt,DE,mn,de,kw OR 'outpatient cost*':ti,ab,tn,tt,DE,mn,de,kw OR 'clinic visit*':ti,ab,tn,tt,DE,mn,de,kw OR 'hospital visit*':ti,ab,tn,tt,DE,mn,de,kw OR 'average wage*':ti,ab,tn,tt,DE,mn,de,kw | economic aspect[tw] OR financial aspect[tw] OR cost of care[tw] OR costs of care[tw] OR direct cost*[tw] OR drug cost*[tw] OR medical cost*[tw] OR physician cost*[tw] OR nurse cost*[tw] OR inpatient cost*[tw] OR outpatient cost*[tw] OR clinic visit*[tw] OR hospital visit*[tw] OR average wage*[tw] | (“economic aspect”):ti,ab,kw OR (“financial aspect”):ti,ab,kw OR (“cost of care”):ti,ab,kw OR (“costs of care”):ti,ab,kw OR (“costing of care”):ti,ab,kw OR (“direct cost”):ti,ab,kw OR (“direct costs”):ti,ab,kw OR (“direct costing”):ti,ab,kw OR (“drug cost”):ti,ab,kw OR (“drug costs”):ti,ab,kw OR (“drug costing”):ti,ab,kw OR (“medical cost”):ti,ab,kw OR (“medical costs”):ti,ab,kw OR (“medical costing”):ti,ab,kw OR (“physician cost”):ti,ab,kw OR (“physician costs”):ti,ab,kw OR (“physician costing”):ti,ab,kw OR (“nurse cost”):ti,ab,kw OR (“nurse costs”):ti,ab,kw OR (“nurse costing”):ti,ab,kw OR (“inpatient cost”):ti,ab,kw OR (“inpatient costs”):ti,ab,kw OR (“outpatient cost”):ti,ab,kw OR (“outpatient costs”):ti,ab,kw OR (“clinic visit”):ti,ab,kw OR (“clinic visits”):ti,ab,kw OR (“clinic visiting”):ti,ab,kw OR (“clinic visitation”):ti,ab,kw OR (“hospital visit”):ti,ab,kw OR (“hospital visits”):ti,ab,kw OR (“hospital visiting”):ti,ab,kw OR (“hospital visitation”):ti,ab,kw OR (“average wage”):ti,ab,kw OR (“average wages”):ti,ab,kw | 285,734 | 60,229 | 13,666 |
| #5 | 'length of stay'/de OR 'length of stay':ti,ab,tn,tt,DE,mn,de,kw OR 'hospitali*ation period':ti,ab,tn,tt,DE,mn,de,kw | length of stay[mesh:noexp] OR length of stay[tw] OR hospitalization period[tw] OR hospitalisation period[tw] | MeSH descriptor: [Length of Stay] this term only OR (“length of stay”):ti,ab,kw OR (“hospitalization period”):ti,ab,kw OR (“hospitalisation period”):ti,ab,kw | 256,167 | 140,411 | 22,082 |
| #6 | 'caregiver burden'/de OR 'caregiver'/de OR 'medical leave'/de OR 'health care financing'/de OR 'drug cost'/de OR 'medical fee'/de OR 'pharmacy fee'/de OR 'hospital charge'/de OR 'work disability'/de OR 'absenteeism'/de OR 'productivity'/de | caregiver burden[mesh:noexp] OR caregivers[mesh:noexp] OR sick leave[mesh:noexp] OR healthcare financing[mesh:noexp] OR health expenditures[mesh:noexp] OR drug costs[mesh:noexp] OR fees, medical[mesh:noexp] OR fees, pharmaceutical[mesh:noexp] OR hospital charges[mesh:noexp] OR return to work[mesh:noexp] OR absenteeism[mesh:noexp] OR efficiency[mesh:noexp] | MeSH descriptor: [Caregiver Burden] this term only OR MeSH descriptor: [Caregivers] this term only OR MeSH descriptor: [Sick Leave] this term only OR MeSH descriptor: [Healthcare Financing] this term only OR MeSH descriptor: [Health Expenditures] this term only OR MeSH descriptor: [Drug Costs] this term only OR MeSH descriptor: [Fees, Medical] this term only OR MeSH descriptor: [Fees, Pharmaceutical] this term only OR MeSH descriptor: [Hospital Charges] this term only OR MeSH descriptor: [Return to Work] this term only OR MeSH descriptor: [Absenteeism] this term only OR MeSH descriptor: [Efficiency] this term only | 290,376 | 125,255 | 5,137 |
| #7 | 'economic burden':ti,ab,tn,tt,DE,mn,de,kw OR 'indirect cost*':ti,ab,tn,tt,DE,mn,de,kw OR 'productivity':ti,ab,tn,tt,DE,mn,de,kw OR 'societal burden':ti,ab,tn,tt,DE,mn,de,kw OR 'societal cost*':ti,ab,tn,tt,DE,mn,de,kw OR 'resource utili*':ti,ab,tn,tt,DE,mn,de,kw OR 'resource us*':ti,ab,tn,tt,DE,mn,de,kw OR 'workdays lost':ti,ab,tn,tt,DE,mn,de,kw OR 'school days lost':ti,ab,tn,tt,DE,mn,de,kw | economic burden[tw] OR indirect cost*[tw] OR productivity[tw] OR societal burden[tw] OR societal cost*[tw] OR resource utili*[tw] OR resource use[tw] OR resource usage[tw] OR workdays lost[tw] OR (school[tw] AND days lost[tw]) | (“economic burden”):ti,ab,kw OR (“indirect cost”):ti,ab,kw OR (“indirect costs”):ti,ab,kw OR (“indirect costing”):ti,ab,kw OR (productivity):ti,ab,kw OR (“societal burden”):ti,ab,kw OR (“societal cost”):ti,ab,kw OR (“societal costs”):ti,ab,kw OR (“societal costing”):ti,ab,kw OR (“resource utilization”):ti,ab,kw OR (“resource utilisation”):ti,ab,kw OR (“resource utility”):ti,ab,kw OR (“resource utilizing”):ti,ab,kw OR (“resource use”):ti,ab,kw OR (“resource usage”):ti,ab,kw OR (“workdays lost”):ti,ab,kw OR (“school days lost”):ti,ab,kw | 168,789 | 111,880 | 10,476 |
| #8 | singapore:ti,ab,tn,tt,DE,mn,de,kw | Singapore[tw] | (singapore):ti,ab,kw | 33,691 | 22,333 | 1,681 |
| #9 | #1 OR #2 | | | 38,415 | 26,934 | 805 |
| #10 | #3 OR #4 OR #5 OR #6 OR #7 | | | 1,431,006 | 596,090 | 63,566 |
| #11 | #9 AND #10 | | | 1,917 | 640 | 26 |
| #12 | #8 AND #11 | | | 63 | 22 | 1 |
| #13 | #12, filters: humans, English, 2009–2022 | | | 47 | 18 | 1 |
