# Supplementary material for: The epidemiologic and economic burden of dengue in Singapore: A systematic review
Source: PLoS Negl Trop Dis. 2024 Jun 10;18(6):e0012240. doi: 10.1371/journal.pntd.0012240 (PMC11192419; doi:10.1371/journal.pntd.0012240)
Supplement: S3 Table — (DOCX) [file pntd.0012240.s003.docx]

**S3 Table.** International and national research databases searched.

| **Database** | **Access link** |
| --- | --- |
| Word Health Organization Publications | <https://www.who.int/publications/i> |
| World Health Organization Publications – Western Pacific | <https://www.who.int/westernpacific/publications/i> |
| World Health Organization Bulletins and Newsletters | <http://apps.who.int/iris/handle/10665/197909> |
| Western Pacific Surveillance and Response Journal | <https://ojs.wpro.who.int/ojs/index.php/wpsar/search> |
| Ministry of Health (Singapore) | <https://www.moh.gov.sg/resources-statistics> |
| National Environment Agency (Singapore) | <https://www.nea.gov.sg/dengue-zika/dengue/quarterly-dengue-surveillance-data> |
| National Centre for Infectious Diseases (Singapore) | <https://www.ncid.sg/Health-Professionals/ncid-research-publications/Pages/default.aspx> |
| National University of Singapore Libraries | <https://myaces.nus.edu.sg/LibOneSearch/search> |
