# Supplementary material for: The epidemiologic and economic burden of dengue in Singapore: A systematic review
Source: PLoS Negl Trop Dis. 2024 Jun 10;18(6):e0012240. doi: 10.1371/journal.pntd.0012240 (PMC11192419; doi:10.1371/journal.pntd.0012240)
Supplement: S4 Table — (DOCX) [file pntd.0012240.s004.docx]

**S4 Table.** Questions in the quality assessment tool for epidemiological studies.

| **Domain** | **Item: *ideal answer* [points]**^a^ | | |
| --- | --- | --- | --- |
|  | **Cross-sectional**^b^ | **Cohort**^c^ | **Case-control**^c^ |
| Selection | Representativeness of the sample:  *truly (all subjects or random sampling) OR somewhat (non-random sampling) representative of the community* [1] | Representativeness of the exposed cohort:  *truly OR somewhat representative of the community* [1] | Is the case definition adequate:  *yes, with independent validation* [1] |
|  | Sample size:  *justified and satisfactory* [1] | Selection of the non-exposed cohort:  *drawn from the same community as the exposed cohort* [1] | Representativeness of the cases:  *consecutive or obviously representative series of cases* [1] |
|  | Ascertainment of exposure:  *validated measurement tool OR non-validated measurement tool, but the tool is available or described* [1] | Ascertainment of exposure:  *secure record (eg surgical records) OR structured interview* [1] | Selection of controls:  *community controls* [1] |
|  | Non-respondents:  *comparability between respondents’ and non-respondents’ characteristics is established, and the response rate is satisfactory* [1] | Demonstration that outcome of interest was not present at start of study:  *yes* [1] | Definition of controls:  *no history of disease (endpoint)* [1] |
| Comparability | The subjects in different outcome groups are comparable, based on the study design or analysis. Confounding factors are controlled:  *study controls for most important factor* [1] *OR for at least one additional factor* [2] | Comparability of cohorts on the basis of the design or analysis:  *study controls for most important factor* [1] *OR for any additional factor* [2] | Comparability of cases and controls on the basis of the design or analysis:  *study controls for most important factor [1] OR for any additional factor* [2] |
| Outcome | Assessment of outcome:  *independent blind assessment OR record linkage OR self-report* [1] | Assessment of outcome:  *independent blind assessment OR record linkage* [1] | Ascertainment of exposure:  *secure record (e.g., surgical records) OR structured interview where blind to case/control status* [1] |
|  | Statistical test:  *the statistical test used to analyze the data is clearly described and appropriate, and the measurement of the association is presented, including confidence intervals or probability level (p-value)* [1] | Was follow-up long enough for outcomes to occur:  *yes* [1] | Same method of ascertainment for cases and controls:  *yes* [1] |
|  |  | Adequacy of follow-up of cohorts:  *complete follow-up (all subjects accounted for) OR subjects lost to follow-up unlikely to introduce bias (small number or % lost, or description provided of those lost)* [1] | Non-response rate:  *same rate for both groups* [1] |
| **Maximum total points** | 8 | 9 | 9 |

^a^Ideal answers are given a maximum of 2 points for the comparability item and 1 point each for the rest.

^b^Adapted from Moskalewicz & Oremus (2020) [21].

^c^Adapted from Wells et al. (2021) [20].
