# Supplementary material for: The epidemiologic and economic burden of dengue in Singapore: A systematic review
Source: PLoS Negl Trop Dis. 2024 Jun 10;18(6):e0012240. doi: 10.1371/journal.pntd.0012240 (PMC11192419; doi:10.1371/journal.pntd.0012240)
Supplement: S5 Table — (DOCX) [file pntd.0012240.s005.docx]

**S5 Table.** Questions in the quality assessment tool for economic studies.^a,b^

| 1. What was the motivation of the study?  2. What was the perspective of the study?  3. Was the appropriate epidemiologic approach taken?  4. Was the study question well specified?  5. Were all relevant, non-trivial cost components and their stakeholders identified?  6. Were necessary timeframes specified?  7. Was a case of disease or risk factor adequately and appropriately defined?  8. Was the counterfactual population occurrence plausible and meaningful?  9. Was an appropriate method(s) of quantification used, such that additional, or excess, costs were measured?  10. Was an appropriate method(s) of quantification used, such that only costs specific to (caused by) the health problem were included (confounders controlled)?  11. Was an appropriate method(s) of quantification used, such that all important effects were captured?  12. Was an appropriate method(s) of quantification used, such that important differences across subpopulations were accounted for?  13. Was an appropriate method(s) of quantification used, such that the required level of detail could be provided?  14. Was the resource quantification method(s) well executed?  15. For population-based studies, were cost allocation methods, data and assumptions valid?  16. For person-based studies, were appropriate statistical tests performed and reported?  17. Were data representative of the study population?  18. Were there any other relevant resource quantification issues?  19. Were healthcare resources valued appropriately?  20. Was the approach for valuing production losses justified, and assumptions valid?  21. Was the inclusion of intangible costs appropriate?  22. Was double counting of mortality-related production losses avoided?  23. Were losses valued appropriately, given the study’s perspective?  24. Did the analysis address the study question?  25. Was a range of estimates presented?  26. Were the main uncertainties identified?  27. Was a sensitivity analysis performed on important (uncertain) parameter estimates?  28. Was a sensitivity analysis performed on key assumptions? (including the counterfactual)  29. Was a sensitivity analysis performed on point estimates? (based on confidence or credible intervals)  30. Was adequate documentation and justification given for cost components, data and sources, assumptions and methods?  31. Was uncertainty around the estimates and its implications adequately discussed?  32. Were important limitations discussed regarding the cost components, data, assumptions and methods?  33. Were the results presented at the appropriate level of detail to answer the study question (cost components; disease subtypes, severity, stage; subpopulation groups; cost bearers)? |
| --- |

Adapted from Larg and Moss (2011) [22].

^a^Responses were given a score of 1 for ‘Yes’, -1 for ‘No’, and 0 for ‘N/A’.

^b^Scores were summed then divided by the total number of items with non-zero score.
