# Supplementary material for: The epidemiologic and economic burden of dengue in Singapore: A systematic review
Source: PLoS Negl Trop Dis. 2024 Jun 10;18(6):e0012240. doi: 10.1371/journal.pntd.0012240 (PMC11192419; doi:10.1371/journal.pntd.0012240)
Supplement: S6 Table — (DOCX) [file pntd.0012240.s006.docx]

**S6 Table.** Incidence rate of dengue by severity from 2000 to 2022 in Singapore.

| **Year** | **Dengue fever** | | **Dengue hemorrhagic fever** | | **Overall** | |
| --- | --- | --- | --- | --- | --- | --- |
|  | **Cases,**  **n (%)**^a^ | **IR,**  **per 100,000 p/y** | **Cases,**  **n (%)**^a^ | **IR,**  **per 100,000 p/y** | **Cases,**  **N** | **IR,**  **per 100,000 p/y**^b^ |
| 2000 | 663 (98.5) | 16.5 | 10 (1.5) | 0.2 | 673 | 16.7 |
| 2001 | 2,366 (99.7) | 57.2 | 6 (0.3) | 0.1 | 2,372 | 57.3 |
| 2002 | 3,937 (99.8) | 94.3 | 8 (0.2) | 0.2 | 3,945 | 94.5 |
| 2003 | NR | -- | NR | -- | 4,788 | 116.4 |
| 2004 | 9,291 (98.2) | 223.0 | 168 (1.8) | 4.0 | 9,459 | 227.0 |
| 2005 | 13,816 (97.2) | 323.9 | 393 (2.8) | 9.2 | 14,209 | 333.1 |
| 2006 | 3,051 (97.6) | 69.3 | 76 (2.4) | 1.7 | 3,127 | 71.0 |
| 2007 | 8,637 (97.9) | 188.2 | 189 (2.1) | 4.1 | 8,826 | 192.3 |
| 2008 | 6,947 (98.8) | 143.6 | 84 (1.2) | 1.7 | 7,031 | 145.3 |
| 2009 | 4,451 (99.0) | 89.3 | 46 (1.0) | 0.9 | 4,497 | 90.2 |
| 2010 | 5,329 (99.4) | 104.9 | 34 (0.6) | 0.7 | 5,363 | 105.6 |
| 2011 | 5,308 (99.6) | 102.4 | 22 (0.4) | 0.4 | 5,330 | 102.8 |
| 2012 | 4,602 (99.4) | 86.6 | 30 (0.6) | 0.6 | 4,632 | 87.2 |
| 2013 | 22,077 (99.6) | 408.9 | 93 (0.4) | 1.7 | 22,170 | 410.6 |
| 2014 | 18,306 (99.9) | 334.6 | 20 (0.1) | 0.4 | 18,326 | 335.0 |
| 2015 | 11,282 (99.9) | 203.8 | 12 (0.1) | 0.2 | 11,294 | 204.0 |
| 2016 | 13,061 (99.8) | 233.0 | 24 (0.2) | 0.4 | 13,085 | 233.4 |
| 2017 | 2,750 (99.4) | 49.0 | 17 (0.6) | 0.3 | 2,767 | 49.3 |
| 2018 | 3,257 (99.2) | 57.7 | 26 (0.8) | 0.5 | 3,283 | 58.2 |
| 2019 | 15,910 (99.4) | 278.9 | 88 (0.6) | 1.5 | 15,998 | 280.5 |
| 2020 | 35,261 (99.8) | 620.2 | 54 (0.2) | 0.9 | 35,315 | 621.1 |
| 2021 | 5,245 (99.8) | 96.2 | 13 (0.2) | 0.2 | 5,258 | 96.4 |
| 2022 | 32,130 (99.9) | 570.0 | 45 (0.1) | 0.8 | 32,175 | 570.8 |

IR, incidence rate; NA, not applicable; NR, not reported; p/y, person-years.

Data retrieved from the Ministry of Health and National Environment Agency (Singapore) [55–57].

^a^Computed as n/N.

^b^Sum of IRs for dengue fever and dengue hemorrhagic fever may not equal overall IR due to rounding.
