# Supplementary material for: The epidemiologic and economic burden of dengue in Singapore: A systematic review
Source: PLoS Negl Trop Dis. 2024 Jun 10;18(6):e0012240. doi: 10.1371/journal.pntd.0012240 (PMC11192419; doi:10.1371/journal.pntd.0012240)
Supplement: S7 Table — (DOCX) [file pntd.0012240.s007.docx]

**S7 Table.** Incidence rate of dengue by age group from 2000 to 2018 in Singapore.

| **Year** | **Incidence rate, per 100,000 person-years** | | | | | | |
| --- | --- | --- | --- | --- | --- | --- | --- |
|  | **0–4 years** | **5–14 years** | **15–24 years** | **25–34 years** | **35–44 years** | **45–54 years** | **≥55 years** |
| 2000 | 2.3 | 3.7 | 16 | 9 | 11.5 | 9.8 | 9.3 |
| 2001 | 9.4 | 30 | 59.8 | 61 | 51.1 | 50.2 | 42.7 |
| 2002 | 10.5 | 58.6 | 122.8 | 96.7 | 92.8 | 75.2 | 60 |
| 2003 | 22.3 | 77.7 | 138.4 | 128.1 | 126.5 | 106.9 | 86.7 |
| 2004 | 49.9 | 189.1 | 322.6 | 279.8 | 241 | 171.5 | 133.7 |
| 2005 | 88.4 | 329.8 | 469.6 | 369.9 | 353.2 | 256.5 | 209.5 |
| 2006 | 18.1 | 42.7 | 67 | 66.6 | 66.8 | 52 | 91.3 |
| 2007 | 48 | 102.6 | 176.7 | 188.8 | 219.7 | 174.4 | 228.9 |
| 2008 | 43.6 | 76.8 | 145.7 | 152.8 | 164.4 | 120.4 | 156.9 |
| 2009 | 22.6 | 43.1 | 97.9 | 90.6 | 91.8 | 77.1 | 100.5 |
| 2010 | 29.5 | 64.7 | 105.5 | 105.6 | 132.8 | 94 | 85.3 |
| 2011 | 32 | 66.2 | 105.9 | 100.3 | 112.3 | 102.7 | 105.4 |
| 2012 | 17 | 42.8 | 91 | 91.7 | 97.5 | 77.3 | 87.3 |
| 2013 | 70.1 | 262 | 527.2 | 483.8 | 476.4 | 392.2 | 297.9 |
| 2014 | 80.5 | 244.2 | 415.3 | 399.9 | 389.2 | 321.3 | 205.8 |
| 2015 | 42.1 | 132.3 | 238.4 | 232.3 | 221.2 | 201.7 | 158.7 |
| 2016 | 48.5 | 163.4 | 275.7 | 263.3 | 264.4 | 246.2 | 182.1 |
| 2017 | 12.3 | 24.8 | 43.3 | 48.8 | 49.6 | 46.7 | 52.8 |
| 2018 | 21 | 37.2 | 64.5 | 64.4 | 60.8 | 64.9 | 60.0 |

Data retrieved from the Ministry of Health (Singapore) [55,56].
