# Supplementary material for: The epidemiologic and economic burden of dengue in Singapore: A systematic review
Source: PLoS Negl Trop Dis. 2024 Jun 10;18(6):e0012240. doi: 10.1371/journal.pntd.0012240 (PMC11192419; doi:10.1371/journal.pntd.0012240)
Supplement: S8 Table — (DOCX) [file pntd.0012240.s008.docx]

**S8 Table.** Incidence rate of dengue by serotype from 2000 to 2022 in Singapore.

| **Year** | **DENV-1** | | **DENV-2** | | **DENV-3** | | **DENV-4** | |
| --- | --- | --- | --- | --- | --- | --- | --- | --- |
|  | **% of samples tested** | **IR,**  **per 100,000 p/y** | **% of samples tested** | **IR,**  **per 100,000 p/y** | **% of samples tested** | **IR,**  **per 100,000 p/y** | **% of samples tested** | **IR,**  **per 100,000 p/y** |
| 2000 | 36.4 | 6.1 | 36.4 | 6.1 | 9.1 | 1.5 | 18.2 | 3.0 |
| 2001 | 7.0 | 4.0 | 79.1 | 45.3 | 0.0 | 0.0 | 14.0 | 8.0 |
| 2002 | 30.6 | 28.9 | 54.1 | 51.2 | 3.8 | 3.6 | 11.5 | 10.8 |
| 2003 | 9.0 | 10.5 | 80.1 | 93.3 | 4.8 | 5.6 | 6.0 | 7.0 |
| 2004 | 67.0 | 152.1 | 27.6 | 62.7 | 2.4 | 5.4 | 3.0 | 6.8 |
| 2005 | 71.2 | 237.3 | 9.2 | 30.6 | 18.9 | 63.0 | 0.6 | 2.1 |
| 2006 | 83.2 | 59.0 | 6.2 | 4.4 | 6.7 | 4.8 | 3.9 | 2.8 |
| 2007 | 6.4 | 12.3 | 88.3 | 169.9 | 4.6 | 8.8 | 0.7 | 1.3 |
| 2008 | 24.6 | 35.8 | 64.6 | 93.8 | 9.8 | 14.2 | 1.0 | 1.5 |
| 2009 | 19.0 | 17.1 | 54.6 | 49.2 | 19.6 | 17.7 | 6.9 | 6.2 |
| 2010 | 15.6 | 16.5 | 73.0 | 77.1 | 7.8 | 8.2 | 3.6 | 3.8 |
| 2011 | 10.5 | 10.8 | 77.1 | 79.3 | 8.6 | 8.8 | 3.8 | 3.9 |
| 2012 | 19.4 | 16.9 | 74.1 | 64.6 | 5.7 | 5.0 | 0.8 | 0.7 |
| 2013 | 61.8 | 253.8 | 24.6 | 101.0 | 11.6 | 47.6 | 2.0 | 8.2 |
| 2014 | 79.3 | 265.7 | 18.0 | 60.2 | 2.5 | 8.4 | 0.2 | 0.7 |
| 2015 | 43.2 | 88.1 | 44.5 | 90.8 | 11.2 | 22.8 | 1.1 | 2.2 |
| 2016 | 29.0 | 67.7 | 51.0 | 119.0 | 18.9 | 44.1 | 1.1 | 2.6 |
| 2017 | 21.5 | 10.6 | 45.5 | 22.4 | 23.7 | 11.7 | 9.3 | 4.6 |
| 2018 | 20.4 | 11.9 | 47.8 | 27.8 | 27.2 | 15.9 | 4.5 | 2.6 |
| 2019 | 4.6 | 12.9 | 63.7 | 178.5 | 24.8 | 69.4 | 7.0 | 19.6 |
| 2020 | 5.3 | 32.6 | 44.4 | 276.0 | 37.4 | 232.0 | 13.0 | 80.6 |
| 2021 | 0.2 | 0.2 | 25.5 | 24.6 | 63.9 | 61.6 | 10.4 | 10.0 |
| 2022 | 3.7 | 21.4 | 16.6 | 94.7 | 77.5 | 442.5 | 2.2 | 12.3 |

DENV, dengue virus; IR, incidence rate; p/y, person-years.

Data retrieved from the Ministry of Health and National Environment Agency (Singapore) [56,57].
