# Supplementary material for: The epidemiologic and economic burden of dengue in Singapore: A systematic review
Source: PLoS Negl Trop Dis. 2024 Jun 10;18(6):e0012240. doi: 10.1371/journal.pntd.0012240 (PMC11192419; doi:10.1371/journal.pntd.0012240)
Supplement: S9 Table — (DOCX) [file pntd.0012240.s009.docx]

**S9 Table.** Characteristics and results of published studies reporting dengue serotype distribution in Singapore.

| **Study identifier** | **Study design** | **Population** | **Year of data collection** | **Samples tested, N** | | **% of N**^a^ | | | |
| --- | --- | --- | --- | --- | --- | --- | --- | --- | --- |
|  |  |  |  |  |  | **DENV-1** | **DENV-2** | **DENV-3** | **DENV-4** |
| Koh 2008 [32] | Retrospective cohort | Cases of dengue reported to MOH | 2000 | | 327 | 36.4 | 36.4 | 9.1 | 18.2 |
|  |  |  | 2001 | | 354 | 6.7 | 80.0 | 0.0 | 13.3 |
|  |  |  | 2002 | | 331 | 30.8 | 53.8 | 3.8 | 11.5 |
|  |  |  | 2003 | | 525 | 9.2 | 80.5 | 4.6 | 5.7 |
|  |  |  | 2004 | | 560 | 67.0 | 27.6 | 2.5 | 3.0 |
|  |  |  | 2005 | | 1,948 | 71.2 | 9.20 | 19 | 0.6 |
| Ler 2011 [34] | Cross-sectional | Volunteer residents from largest clusters of infection | 2007 | | 1,044 | 6.4 | 88.3 | 4.6 | 0.7 |
| Thein 2012 [58] | Prospective cohort | Adult febrile patients | 2005–2011 | | 345 | 25.2 | 49.6 | 21.2 | 4.1 |
| Arima 2013 [29] | Retrospective cohort | Cases of dengue reported to MOH | 2011 | | 712 | 10.0 | 77.0 | 9.0 | 4.0 |
| Yung 2015 [53] | Prospective cohort | Adult febrile patients | 2005 | | 134 | 49.3 | 3.7 | 47.0 | NR |
|  |  |  | 2006 | | 4 | 75.0 | 25.0 | 0.0 | NR |
|  |  |  | 2007 | | 46 | 0.0 | 100.0 | 0.0 | NR |
|  |  |  | 2008 | | 23 | 26.1 | 73.9 | 0.0 | NR |
|  |  |  | 2009 | | 11 | 0.0 | 72.7 | 27.3 | NR |
|  |  |  | 2010 | | 121 | 15.7 | 77.7 | 6.6 | NR |
|  |  |  | 2011 | | 112 | 8.0 | 86.6 | 5.4 | NR |
| Hapuarachchi 2016 [31] | Retrospective cohort | Cases of dengue reported to MOH | 2013 | | 8,216 | 61.7 | 24.8 | 11.5 | 2.0 |
|  |  |  | 2014 | | 6,598 | 79.2 | 18.1 | 2.5 | 0.2 |
| Sadarangani 2016 [43] | Retrospective cohort | Hospitalized patients with confirmed dengue | 2015 | | 13 | 38.5 | 61.5 | 0.0 | 0.0 |
| Rouers 2021 [42] | Prospective cohort | Adult patients with suspected dengue and fever <6 days, recruited at a hospital | NR | | 50 | 42.0 | 46.0 | 8.0 | 4.0 |

DENV, dengue virus; MOH, Ministry of Health; NR, not reported.

^a^Percentages directly lifted from source, if available, or otherwise computed; totals may not equal 100% due to rounding.
