# Supplementary material for: The epidemiologic and economic burden of dengue in Singapore: A systematic review
Source: PLoS Negl Trop Dis. 2024 Jun 10;18(6):e0012240. doi: 10.1371/journal.pntd.0012240 (PMC11192419; doi:10.1371/journal.pntd.0012240)
Supplement: S10 Table — (DOCX) [file pntd.0012240.s010.docx]

**S10 Table.** Dengue hospitalization rate by age group from 2003 to 2017 in Singapore.

| **Year** | **Hospitalization rate, %** | | | | | | | |
| --- | --- | --- | --- | --- | --- | --- | --- | --- |
|  | **0–14**  **Years** | **15–24**  **years** | **25–34**  **years** | **35–44**  **years** | **45–54**  **years** | **55–64**  **years** | **≥65**  **years** | **Overall** |
| 2003 | 87.6 | 97.4 | 96.2 | 100 | 100 | 100 | 100 | 97.3 |
| 2004 | 77.7 | 89.2 | 84.5 | 89.3 | 100 | 100 | 99.4 | 88.8 |
| 2005 | 83.4 | 100 | 96.1 | 97.3 | 100 | 100 | 100 | 96.3 |
| 2006 | 81 | 86.9 | 77.2 | 80.9 | 81.3 | 60.7 | 35.3 | 72.6 |
| 2007 | 44 | 55.9 | 56.5 | 59.3 | 67.7 | 69.7 | 50.4 | 58.1 |
| 2008 | 46.7 | 45.9 | 42.7 | 48.1 | 55 | 56.7 | 40 | 47 |
| 2009 | 50.7 | 46.5 | 44.9 | 53.8 | 57.1 | 58.7 | 49.9 | 50.6 |
| 2010 | 46.4 | 44.6 | 43.2 | 44.6 | 58.2 | 59.6 | 52.8 | 47.9 |
| 2011 | 41.8 | 40.3 | 36.3 | 41.1 | 49.3 | 56.7 | 45.4 | 43 |
| 2012 | 41 | 37.7 | 34.5 | 43.7 | 47.7 | 55.1 | 45.3 | 41.9 |
| 2013 | 33.8 | 27.9 | 24.1 | 29.1 | 38.5 | 46.5 | 49.4 | 31.6 |
| 2014 | 27.5 | 20.8 | 19.9 | 22.4 | 29.6 | 43.8 | 48.1 | 25.6 |
| 2015 | 31.9 | 22.9 | 22.7 | 26.1 | 36.4 | 42.7 | 55.4 | 29.9 |
| 2016 | 25.6 | 21.8 | 20.4 | 25.9 | 32.5 | 45.4 | 55.4 | 28.6 |
| 2017 | 30.6 | 32.6 | 27.3 | 34.9 | 38.5 | 46.9 | 47.2 | 35.9 |

Adapted from Ang 2019 [28].
