# Supplementary material for: The epidemiologic and economic burden of dengue in Singapore: A systematic review
Source: PLoS Negl Trop Dis. 2024 Jun 10;18(6):e0012240. doi: 10.1371/journal.pntd.0012240 (PMC11192419; doi:10.1371/journal.pntd.0012240)
Supplement: S11 Table — (DOCX) [file pntd.0012240.s011.docx]

**S11 Table.** Dengue case fatality rate from 2000 to 2022 in Singapore.

| **Year** | **Deaths, n** | **Cases, N** | **Case fatality rate, %**^a^ **(95% confidence interval)** |
| --- | --- | --- | --- |
| 2000 | 2 | 673 | 0.30 (0.04, 1.07) |
| 2001 | 4 | 2,372 | 0.17 (0.05, 0.43) |
| 2002 | 4 | 3,945 | 0.10 (0.03, 0.26) |
| 2003 | 6 | 4,788 | 0.13 (0.06, 0.27) |
| 2004 | 9 | 9,459 | 0.10 (0.05, 0.18) |
| 2005 | 27 | 14,209 | 0.19 (0.13, 0.28) |
| 2006 | 10 | 3,127 | 0.32 (0.17, 0.59) |
| 2007 | 24 | 8,826 | 0.27 (0.18, 0.40) |
| 2008 | 10 | 7,031 | 0.14 (0.08, 0.26) |
| 2009 | 8 | 4,497 | 0.18 (0.09, 0.35) |
| 2010 | 6 | 5,363 | 0.11 (0.05, 0.24) |
| 2011 | 6 | 5,330 | 0.11 (0.05, 0.25) |
| 2012 | 2 | 4,632 | 0.04 (0.01, 0.16) |
| 2013 | 8 | 22,170 | 0.04 (0.02, 0.07) |
| 2014 | 6 | 18,326 | 0.03 (0.02, 0.07) |
| 2015 | 6 | 11,294 | 0.05 (0.02, 0.12) |
| 2016 | 12 | 13,085 | 0.09 (0.05, 0.16) |
| 2017 | 2 | 2,767 | 0.07 (0.01, 0.26) |
| 2018 | 6 | 3,283 | 0.18 (0.08, 0.40) |
| 2019 | 20 | 15,998 | 0.13 (0.08, 0.19) |
| 2020 | 32 | 35,315 | 0.09 (0.06, 0.13) |
| 2021 | 5 | 5,258 | 0.10 (0.04, 0.22) |
| 2022 | 19 | 32,175 | 0.06 (0.04, 0.09) |

Data retrieved from the Ministry of Health and National Environment Agency (Singapore) [55,56].

^a^Computed as n/N.
