# Supplementary material for: The epidemiologic and economic burden of dengue in Singapore: A systematic review
Source: PLoS Negl Trop Dis. 2024 Jun 10;18(6):e0012240. doi: 10.1371/journal.pntd.0012240 (PMC11192419; doi:10.1371/journal.pntd.0012240)
Supplement: S12 Table — (DOCX) [file pntd.0012240.s012.docx]

**S12 Table.** Dengue case fatality rate by disease severity for select years in Singapore.

| **Year** | **Dengue fever** | | | **Dengue hemorrhagic fever** | | |
| --- | --- | --- | --- | --- | --- | --- |
|  | **Deaths, n** | **Cases, N** | **Case fatality rate, %**^a^ **(95% CI)** | **Deaths, n** | **Cases, N** | **Case fatality rate, %**^a^ **(95% CI)** |
| 2002 | 4 | 3,937 | 0.10 (0.03, 0.26) | 0 | 8 | 0.00 (0.00, 36.94) |
| 2005 | 24 | 13,816 | 0.17 (0.12, 0.26) | 3 | 393 | 0.76 (0.16, 2.21) |
| 2006 | 8 | 3,051 | 0.26 (0.13, 0.52) | 2 | 76 | 2.63 (0.32, 9.18) |
| 2007 | 16 | 8,637 | 0.19 (0.11, 0.30) | 8 | 199 | 4.02 (2.05, 7.73) |
| 2008 | 9 | 6,947 | 0.13 (0.07, 0.25) | 1 | 84 | 1.19 (0.03, 6.46) |
| 2009 | 3 | 4,451 | 0.07 (0.01, 0.20) | 5 | 46 | 10.87 (4.73, 23.04) |
| 2010 | 4 | 5,329 | 0.08 (0.02, 0.19) | 2 | 34 | 5.88 (0.72, 19.68) |
| 2011 | 4 | 5,308 | 0.08 (0.02, 0.19) | 2 | 22 | 9.09 (1.12, 29.16) |

Data retrieved from the Ministry of Health (Singapore) [55,56].

^a^Computed as n/N.
