# Supplementary material for: The epidemiologic and economic burden of dengue in Singapore: A systematic review
Source: PLoS Negl Trop Dis. 2024 Jun 10;18(6):e0012240. doi: 10.1371/journal.pntd.0012240 (PMC11192419; doi:10.1371/journal.pntd.0012240)
Supplement: S13 Table — (DOCX) [file pntd.0012240.s013.docx]

**S13 Table.** Characteristics and results of published studies reporting dengue seroprevalence in Singapore.

| **Study identifier** | **Study design** | **Data collection period** | **Population** | **Sample size** | **Age, years**^a^ | **Testing method** | **Seroprevalence,**  **% (95% CI)** | **Stratification variable** | |
| --- | --- | --- | --- | --- | --- | --- | --- | --- | --- |
|  |  |  |  |  |  |  |  | **Stratified seroprevalence, % (95% CI)** | |
| Wilder-Smith 2004 [49] | Cross-sectional | August 2002 | Asymptomatic volunteering staff and visitors to a hospital | 298 | 18–45 | ELISA (IgG) | 45.0 | Age group | |
|  |  |  |  |  |  |  |  | 18–25 | 17.0 |
|  |  |  |  |  |  |  |  | 26–35 | 44.0 |
|  |  |  |  |  |  |  |  | 36–45 | 74.0 |
| Chow 2005 [30] | Cross-sectional | 1998–2000 | Healthy university graduates | 184 | 19–26 | ELISA (IgG) | 22.0 | None | |
| Seet 2005 [44] | Cross-sectional | Mar 2002–Apr 2002 | Chinese migrant workers living within a construction site | 274 | 33 ± 4.6 | ELISA (IgM) | 10.0 | None | |
| Wilder-Smith 2005 [50] | Cross-sectional | August 2002 | Healthy university graduates | 164 | 18–30 | ELISA (IgG), PRNT for serotyping | 29.8 | Serotype | |
|  |  |  |  |  |  |  |  | DENV-1 | 23.8 |
|  |  |  |  |  |  |  |  | DENV-2 | 22.6 |
|  |  |  |  |  |  |  |  | DENV-3 | 26.2 |
|  |  |  |  |  |  |  |  | DENV-4 | 18.3 |
|  |  |  |  |  |  |  |  | Number of serotypes | |
|  |  |  |  |  |  |  |  | 1 | 7.3 |
|  |  |  |  |  |  |  |  | 2 | 1.2 |
|  |  |  |  |  |  |  |  | 3 | 4.3 |
|  |  |  |  |  |  |  |  | 4 | 17.1 |
| Yew 2009 [52] | Cross-sectional | September 2004–December 2004 | Residents with residual sera from a national health survey | 4,152 | 18–74 | ELISA (IgG) | 59.0 (57.5, 60.5) | Age group | |
|  |  |  |  |  |  |  |  | 18–24 | 17.2 |
|  |  |  |  |  |  |  |  | 25–34 | 34.1 |
|  |  |  |  |  |  |  |  | 35–44 | 61.7 |
|  |  |  |  |  |  |  |  | 45–54 | 78.1 |
|  |  |  |  |  |  |  |  | 55–74 | 88.9 |
| Lye 2010 [37] | Retrospective cohort | 2004 | Hospitalized patients with dengue | 1,307 | 17–76 | Rapid immunochromatography | 35.0 | Age group | |
|  |  |  |  |  |  |  |  | <60 | 34.0 |
|  |  |  |  |  |  |  |  | ≥60 | 64.0 |
| Leo 2012 [33] | RCT | April 2009–October 2009 | Healthy individuals without current dengue infection | 585 | 2–45 | PRNT | TG: 26.5 (22.3, 30.9)  CG: 32.4 (24.9, 40.7) | Age group | |
|  |  |  |  |  |  |  |  | 2–11 | TG: 19.6 (13.5, 26.9)  CG: 26.0 (14.6, 40.3) |
|  |  |  |  |  |  |  |  | 12–17 | TG: 13.5 (8.3, 20.2)  CG: 15.2 (6.3, 28.9) |
|  |  |  |  |  |  |  |  | 18–45 | TG: 46.5 (38.1, 55.0)  CG: 55.1 (40.2, 69.3) |
|  |  |  |  |  |  |  |  | Number of serotypes | |
|  |  |  |  |  |  |  |  | ≥1 | TG: 26.5 (22.3, 30.9)  CG: 32.4 (24.9, 40.7) |
|  |  |  |  |  |  |  |  | ≥2 | TG: 15.3 (12.0, 19.1)  CG: 14.5 (9.2, 21.3) |
|  |  |  |  |  |  |  |  | ≥3 | TG: 11.8 (8.9, 15.3)  CG: 11.7 (7.0, 18.1) |
|  |  |  |  |  |  |  |  | 4 | TG: 8.8 (6.3, 11.9)  CG: 7.6 (3.8, 13.2) |
| Yap 2013 [51] | Cross-sectional | 2007 | Residents of dengue outbreak area | 3,939 | 7–85 | ELISA (IgM and IgG) | 65.9 | None | |
| Ang 2015a [26] | Cross-sectional | 17 March 2010–13 June 2010 | Residents with residual sera from a national health survey | 3,293 | 1 –79 | ELISA (IgG) | Crude: 56.8 (55.1, 58.5)  Age-standardized: 54.4 (52.7, 56.2) | Age group | |
|  |  |  |  |  |  |  |  | 18–29 | 17.8 |
|  |  |  |  |  |  |  |  | 30–39 | 42.1 |
|  |  |  |  |  |  |  |  | 40–49 | 60.4 |
|  |  |  |  |  |  |  |  | 50–59 | 77.8 |
|  |  |  |  |  |  |  |  | 60–69 | 92.4 |
|  |  |  |  |  |  |  |  | 70–79 | 92.7 |
| Ang 2015b [27] | Cross-sectional | August 2008–July 2010 | Inpatients (hospitalization or day surgery) without current dengue and with residual sera from a national survey | 1,200 | 1–17 | ELISA (IgG) | 10.4 (8.7, 12.1) | Age group | |
|  |  |  |  |  |  |  |  | 1–6 | 11.0 |
|  |  |  |  |  |  |  |  | 7–12 | 10.0 |
|  |  |  |  |  |  |  |  | 13–17 | 10.3 |
| Low 2015 [35] | Cross-sectional | December 2009–February 2010 | Healthy blood donors | 3,627 | 16–60 | ELISA (IgG), PRNT for serotyping | Crude: 52 (50.3, 53.6)  Age-standardized: 50.8 (49.4, 52.3) | Age group | |
|  |  |  |  |  |  |  |  | 16–20 | 16.1 |
|  |  |  |  |  |  |  |  | 21–25 | 16.4 |
|  |  |  |  |  |  |  |  | 26–30 | 32.8 |
|  |  |  |  |  |  |  |  | 31–35 | 43.0 |
|  |  |  |  |  |  |  |  | 36–40 | 48.4 |
|  |  |  |  |  |  |  |  | 41–45 | 57.3 |
|  |  |  |  |  |  |  |  | 46–50 | 69.6 |
|  |  |  |  |  |  |  |  | 51–55 | 79.6 |
|  |  |  |  |  |  |  |  | 56–60 | 86.6 |
|  |  |  |  |  |  |  |  | Serotype^b^ | |
|  |  |  |  |  |  |  |  | DENV-1 | 9.7–77.9 |
|  |  |  |  |  |  |  |  | DENV-2 | 14.0–86.6 |
|  |  |  |  |  |  |  |  | DENV-3 | 2.7–57.7 |
|  |  |  |  |  |  |  |  | DENV-4 | 2.1–31.8 |
|  |  |  |  |  |  |  |  | Number of serotypes^b^ | |
|  |  |  |  |  |  |  |  | 1 | 5.8–10.1 |
|  |  |  |  |  |  |  |  | 2 | 4.3–21.0 |
|  |  |  |  |  |  |  |  | 3 | 2.7–40.4 |
|  |  |  |  |  |  |  |  | 4 | 0.5–23.1 |
| Tan 2019 [46] | Cross-sectional | December 2013–February 2014 | Healthy blood donors | 3,813 | 16–74 | ELISA (IgG) | Crude: 49.1 (47.6, 50.7)  Age-standardized: 49.8 (48.4, 51.1) | Age group | |
|  |  |  |  |  |  |  |  | 16–20 | 15.3 |
|  |  |  |  |  |  |  |  | 21–25 | 17.6 |
|  |  |  |  |  |  |  |  | 26–30 | 27.6 |
|  |  |  |  |  |  |  |  | 31–35 | 35.7 |
|  |  |  |  |  |  |  |  | 36–40 | 38.2 |
|  |  |  |  |  |  |  |  | 41–45 | 50.5 |
|  |  |  |  |  |  |  |  | 46–50 | 59.6 |
|  |  |  |  |  |  |  |  | 51–55 | 71.5 |
|  |  |  |  |  |  |  |  | 56–60 | 80.1 |
|  |  |  |  |  |  |  |  | >60 | 87.9 |
|  |  | June–August 2017 | Healthy blood donors | 4,002 | 16–74 | ELISA (IgG) | Crude: 45.7 (44.1, 47.2)  Age-standardized: 48.6 (47.0, 50.0) | Age group | |
|  |  |  |  |  |  |  |  | 16–20 | 13.8 |
|  |  |  |  |  |  |  |  | 21–25 | 17.3 |
|  |  |  |  |  |  |  |  | 26–30 | 24.9 |
|  |  |  |  |  |  |  |  | 31–35 | 27.5 |
|  |  |  |  |  |  |  |  | 36–40 | 35.6 |
|  |  |  |  |  |  |  |  | 41–45 | 42.9 |
|  |  |  |  |  |  |  |  | 46–50 | 55.5 |
|  |  |  |  |  |  |  |  | 51–55 | 65.4 |
|  |  |  |  |  |  |  |  | 56–60 | 77.7 |
|  |  |  |  |  |  |  |  | >60 | 85.0 |
| Park 2020 [39] | RCT (follow-up study) | July 2016–February 2017 | same as Leo 2012 | | | | | | |
| Tricou 2020 [48] | RCT | June 2015–September 2017 | Healthy participants without current dengue | 347 | 21–45 | Microneutralization assay | 53.6 | Serotype | |
|  |  |  |  |  |  |  |  | DENV-1 | 48.7 |
|  |  |  |  |  |  |  |  | DENV-2 | 49.0 |
|  |  |  |  |  |  |  |  | DENV-3 | 45.2 |
|  |  |  |  |  |  |  |  | DENV-4 | 41.2 |
| Rouers 2021 [42] | Prospective cohort | NR | Patients with suspected dengue and fever <6 days, recruited at a hospital | 68 | 36.3 | ELISA (IgG) | 55.9 | None | |
| Lu 2022 [36] | Prospective cohort | NR | Community-dwelling individuals with or without current dengue | 844 | ≥55 | ELISA (IgG) | 73.7 | None | |

CG, control group; CI, confidence interval; DENV, dengue virus; ELISA, enzyme-linked immunosorbent assay; IgG, immunoglobulin G; IgM, immunoglobulin M; NR, not reported; PRNT, plaque reduction neutralization test; RCT, randomized controlled trial; TG, treatment group.

^a^Range or mean ± standard deviation.

^b^Range is shown because value depends on age.
