# Supplementary material for: The epidemiologic and economic burden of dengue in Singapore: A systematic review
Source: PLoS Negl Trop Dis. 2024 Jun 10;18(6):e0012240. doi: 10.1371/journal.pntd.0012240 (PMC11192419; doi:10.1371/journal.pntd.0012240)
Supplement: S14 Table — (DOCX) [file pntd.0012240.s014.docx]

**S14 Table.** Characteristics of published studies reporting costs of illness from dengue in Singapore.

| **Study identifier** | **Data sources** | **Parameter** | **Parameter description** | | | **Value** | **Unit** |
| --- | --- | --- | --- | --- | --- | --- | --- |
| Carrasco 2011 [14] | Surveillance data from the Ministry of Health, prospective Early Dengue (EDEN) Infection and Outcomes study, Adult Retrospective Dengue Study at Tan Tock Seng Hospital (ARDENT) (2004–2008), hospital bills | Direct medical costs | Hospital costs per hospitalized case per day | | | 431 ± 597 | 2010 USD |
|  |  |  | Average costs per ambulatory visit | | | 62.1 | 2010 USD |
|  |  | Direct non-medical costs | Transport costs | | | 3.7 | 2010 USD |
|  |  | Indirect costs | Average household service losses per day | | | 35 | 2010 USD |
|  |  |  | Cost of providing primary education per student per day | | | 21 | 2010 USD |
|  |  |  | Cost of providing secondary education per student per day | | | 29.3 | 2010 USD |
|  |  |  | Average productivity loss per absent day of work in individuals 18–64 years old | | | 163 | 2010 USD |
|  |  |  | Annual expenditure on dengue control | | | 50 | 2010 million USD |
|  |  | Other | Number of ambulatory visits | | | 4.33 | Visits |
|  |  |  | Hospital days | | | 4.6–4.8 | Days |
|  |  |  | Sick days (unreported) | | | 4 | Days |
|  |  |  | Sick days (reported) | | | 10.4 | Days |
|  |  |  | Sick days (dengue hemorrhagic fever) | | | 14 | Days |
|  |  | Expansion factor | Ambulatory | Age-dependent symptomatic rate | 15–24 years old | 3.8 | None |
|  |  |  |  |  | 25–34 years old | 13.1 | None |
|  |  |  |  |  | 35–44 years old | 24.3 | None |
|  |  |  |  |  | 45–54 | 45.3 | None |
|  |  |  |  |  | >55 | 50.0 | None |
|  |  |  |  | Constant symptomatic rate | 15–24 years old | 1.7–3.6 | None |
|  |  |  |  |  | 25–34 years old | 3.8–8.2 | None |
|  |  |  |  |  | 35–44 years old | 6.1 –13.4 | None |
|  |  |  |  |  | 45–54 | 11.1–24.2 | None |
|  |  |  |  |  | >55 | 12.2 –26.5 | None |
|  |  |  | Hospitalized | | | 1.4–3.4 | None |
| Soh 2021 [45] | Previous Singapore literature, official sources, consultation with National Environment Agency | Direct medical costs | Average cost per visit (Community Health Assist Scheme) | | | 32.8–56.1 | 2010 USD |
|  |  |  | Average cost per visit (polyclinic) | | | 58–74.8 | 2010 USD |
|  |  |  | Average cost per visit (public hospital) | | | 1780.9–3014 | 2010 USD |
|  |  |  | Average cost per visit (emergency department) | | | 135.3–281.5 | 2010 USD |
|  |  | Direct non-medical costs | Transport cost | | | 3.7 | 2010 USD |
|  |  | Indirect costs | Average household services losses per day | | | 35 | 2010 USD |
|  |  |  | Cost of providing primary education per student per day | | | 21.2–36.6 | 2010 USD |
|  |  |  | Cost of providing secondary education per student per day | | | 29.6–48.5 | 2010 USD |
|  |  |  | Average productivity loss per absent day of work in individuals 18–64 years old | | | 155.4–200 | 2010 USD |
|  |  | Other | Number of ambulatory visits | | | 4.33 | Visits |
|  |  |  | Hospital days | | | 3.2–3.7 | Days |
|  |  |  | Sick days (unreported) | | | 4 | Days |
|  |  |  | Sick days (reported) | | | 10.4 | Days |
|  |  |  | Sick days (dengue hemorrhagic fever) | | | 14 | Days |
|  |  | Expansion factor | Ambulatory | Age-dependent symptomatic rate | | Same as Carrasco 2011 | None |
|  |  |  |  | Constant symptomatic rate | |  |  |
|  |  |  | Hospitalized | | | 1.0 | None |
| Shepard 2013 [12] | Surveillance data from Ministry of Health and World Health Organization, published studies, literature review (1995–2012) to estimate economic burden | Direct costs | Unit costs per hospitalized dengue case | | | 2060.5 | 2010 USD |
|  |  |  | Unit costs per ambulatory dengue case | | | 394.9 | 2010 USD |
|  |  | Indirect costs | Unit costs per hospitalized dengue case | | | 948 | 2010 USD |
|  |  |  | Unit costs per ambulatory dengue case | | | 873.4 | 2010 USD |
|  |  | Expansion factor | Ambulatory | | | 5.0 | None |
|  |  |  | Hospitalized | | | 2.5 | None |
|  |  |  | Total | | | 4.1 | None |
